# Integrative analysis of transcriptomics and clinical data uncovers the tumor-suppressive activity of MITF in prostate cancer

**DOI:** 10.1038/s41419-018-1096-6

**Published:** 2018-10-11

**Authors:** Lorea Valcarcel-Jimenez, Alice Macchia, Natalia Martín-Martín, Ana Rosa Cortazar, Ariane Schaub-Clerigué, Mikel Pujana-Vaquerizo, Sonia Fernández-Ruiz, Isabel Lacasa-Viscasillas, Aida Santos-Martin, Ana Loizaga-Iriarte, Miguel Unda-Urzaiz, Ivana Hermanova, Ianire Astobiza, Mariona Graupera, Julia Starkova, James Sutherland, Rosa Barrio, Ana M. Aransay, Arkaitz Carracedo, Verónica Torrano

**Affiliations:** 1 CIC bioGUNE, Bizkaia Technology Park, 801ª bld, 48160 Derio, Bizkaia Spain; 2 CIBERONC, Madrid, Spain; 3 Department of Urology, Basurto University Hospital, 48013 Bilbao, Spain; 4 Vascular Signalling Laboratory, Institut d´Investigació Biomèdica de Bellvitge (IDIBELL), Gran Via de l’Hospitalet 199-203, Barcelona, Spain; 5 CLIP-Childhood Leukaemia Investigation. Dept. of Pediatric Hematology and Oncology. Second Faculty of Medicine, Charles University, Prague, Czech Republic; 6 Biochemistry and Molecular Biology Department, University of the Basque Country (UPV/EHU), P.O. Box 644, 48080 Bilbao, Spain

## Abstract

The dysregulation of gene expression is an enabling hallmark of cancer. Computational analysis of transcriptomics data from human cancer specimens, complemented with exhaustive clinical annotation, provides an opportunity to identify core regulators of the tumorigenic process. Here we exploit well-annotated clinical datasets of prostate cancer for the discovery of transcriptional regulators relevant to prostate cancer. Following this rationale, we identify Microphthalmia-associated transcription factor (MITF) as a prostate tumor suppressor among a subset of transcription factors. Importantly, we further interrogate transcriptomics and clinical data to refine MITF perturbation-based empirical assays and unveil Crystallin Alpha B (CRYAB) as an unprecedented direct target of the transcription factor that is, at least in part, responsible for its tumor-suppressive activity in prostate cancer. This evidence was supported by the enhanced prognostic potential of a signature based on the concomitant alteration of MITF and CRYAB in prostate cancer patients. In sum, our study provides proof-of-concept evidence of the potential of the bioinformatics screen of publicly available cancer patient databases as discovery platforms, and demonstrates that the MITF-CRYAB axis controls prostate cancer biology.

## Introduction

Balanced integration of intracellular circuits operates within a normal cell to sustain physiological homeostasis. Alterations in some, if not all, of these circuits converge in changes on gene expression, which will eventually enable the acquisition and sustenance of the hallmarks of cancer cells^1^. This event emphasizes the importance of maintaining the transcriptional homeostasis in normal cells and places gene expression deregulation at the core of cancer research interests.

In the last decades, transcriptomics data derived from cancer specimens have become an important resource for the classification, stratification, and molecular driver identification in tumors. We and others have demonstrated that deregulation of gene expression is a key node for cancer pathogenesis and progression^2–6^. Prostate cancer (PCa) research exemplifies the effort in deciphering the genomics and transcriptomics landscape of tumors, and extremely valuable data have been generated^7–13^. In spite of the public availability of these relevant data, they are still underexploited by the scientific community to understand PCa biology. In this regard, the computational tools and dataset selection strategies to carry out these studies are a bottleneck for the cancer research field.

By combining integrated-bioinformatics screening of clinically relevant PCa datasets with in vivo and in vitro molecular biology assays, we have recently described the metastasis suppressor activity of peroxisome proliferator-activated receptor γ (PPARγ) coactivator alpha (PGC1α)^14,15^. This transcriptional coactivator is a major regulator of mitochondrial biogenesis and function, and has an inherent capacity to integrate environmental signals and cellular energetic demands. This ability empowers PGC1α to be a driver in shaping responses to metabolic stress during different physiologic and tumorigenic processes^16^. As might be expected due to its fundamental role in normal and cancer scenarios, the regulation of PGC1α expression, from the genomic to the protein level, is complex and dynamic^17^. At the level of mRNA expression, one of the well-defined direct regulators of PGC1α is the Microphthalmia-associated transcription factor (MITF)^18^.

MITF is a basic helix-loop-helix leucine zipper (bHLHZIP) transcription factor that regulates the expression of lineage commitment programs that are essential for propagation of the melanocyte lineage^19^. The existence of different MITF transcript variants is the result of both alternative splicing and promoter activation that results in the cell-type-specific expression of the different MITF isoforms (A, CX, MC, C, E, H, D, B, M, J)^20^. The melanoma-specific isoform M-MITF is the best studied isoform and, despite some controversy, its expression is generally deregulated in melanoma. Although MITF alone cannot act as a classical oncogene, it has been called a “lineage survival oncogene” for melanoma^19,21^. Importantly, the presence or absence of the M-MITF-PGC1α regulatory axis has stratification potential in melanoma and informs on the efficacy of BRAF inhibitor treatments^18,22^. Although the expression of MITF has been detected in other types of tumors different from melanoma^23,24^, its active role in the progression of these diseases, including PCa, remains unexplored.

Crystallin Alpha-B (CRYAB) is a ubiquitous small heat-shock protein that is expressed in response to a wide range of physiological and nonphysiological conditions preventing aggregations of denatured proteins. In a wide variety of tumor types CRYAB has been found to be overexpressed and associated with disease progression^25–29^ and poor prognosis^30,31^. However, in PCa and nasopharyngeal cancers, CRYAB expression is decreased^32,33^, pointing at possible tumor-suppressive activity of CRYAB in these cancer scenarios.

In the present study, by combining an exhaustive interrogation of seven publically available PCa databases with refined empirical assays, we have identified MITF as a prostate tumor suppressor. In addition, we have unveiled CRYAB as a novel direct target of the transcription factor that is, at least in part, responsible for its tumor-suppressive activity in PCa. Importantly, the tumor-suppressive role for this novel MITF-CRYAB axis is supported by the enhanced prognostic potential of a signature based on the concomitant alteration of both genes in PCa patients.

## Results

### Bioinformatics screening identifies MITF as a transcription factor altered in prostate cancer

**Fig. 1:**
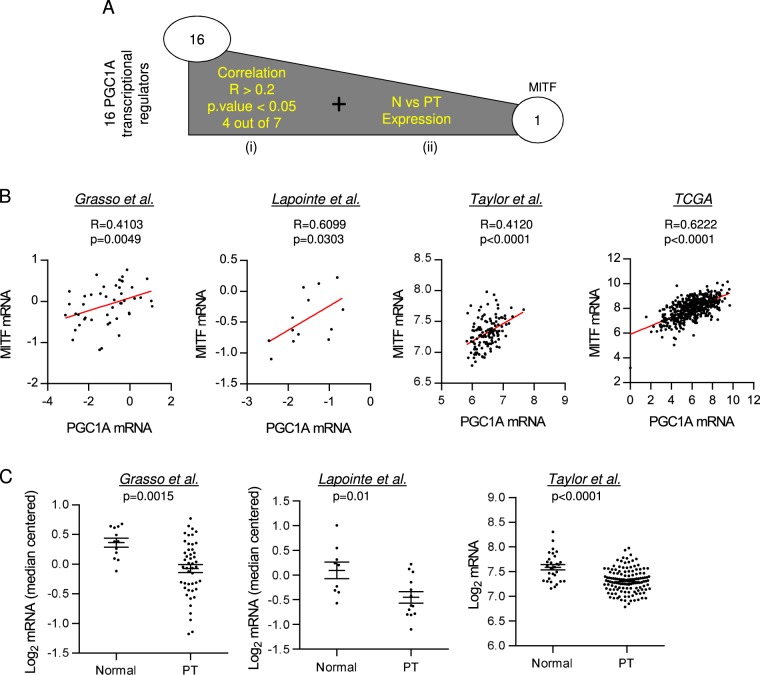
MITF expression correlates with PGC1A expression and is downregulated in PCa. **a** Schematic representation of candidate screening to mediate PGC1A downregulation in PCa. Candidate selection was performed by applying two different selection criteria based on the consistency within the datasets used (>50%): the expression of the candidate must be consistently (i) correlated with the PGC1A’s and (ii) altered in the disease. **b** Correlation analysis between PGC1A and MITF expression in primary tumor (PT) specimens of different PCa datasets (refs. ^9–11^ and TCGA provisional). Sample sizes: Grasso *n* = 45; Lapointe *n* = 13; Taylor *n* = 131 and TCGA provisional *n* = 495. **c** MITF expression in normal prostate and primary tumor (PT) specimens in different datasets^9–11^. Correlation (**b**) and expression (**c**) data from Taylor dataset corresponds to the mean signal of all isoforms of the transcripts. In (**b**) and (**c**), each dot corresponds to an individual specimen. Sample sizes: Grasso et al. (Normal, *n* = 12; PT, *n* = 45); Lapointe et al. (Normal, *n* = 9; PT, *n* = 13); Taylor et al. (Normal, *n* = 29; PT, *n* = 131). Error bars represent s.e.m. Statistic test: Spearman correlation *R* (**b**) and Mann−Whitney test (**c**). *p*, *p*- value

We have recently demonstrated that the reduced expression of the transcriptional coactivator PGC1α is a causal event for metastatic PCa^14^. We sought to identify transcriptional regulators related to the alteration in PGC1α expression. We designed a bioinformatics strategy based on the analysis of 16 genes directly linked to the regulation of *PGC1A* gene^17,22,34–38^, in order to identify transcription factors that could be relevant to PCa biology. For the candidate screen we applied selection criteria based on the consistency of, first, the correlation with *PGC1A* expression and second, the expression of each individual candidate in seven publicly available PCa datasets^7,9–13^ (Fig. 1a). We selected those candidates whose expression in primary tumors correlated with *PGC1A* (*R* ≥ 0.2 and *p* value ≤ 0.05 in more than 50% of the datasets) (Supplementary Figure 1A) and was altered when compared to normal specimens. For genes exhibiting various transcript variants, the correlation analysis was initially performed using the average signal (Supplementary Figure 1A) and, when available (only Taylor dataset^11^), the correlation was confirmed in all the individual isoforms (Supplementary Table 1). The transcription factor MITF was the sole candidate that complied with the established criteria. We observed a consistent correlation between *PGC1A* and *MITF* in four out of the seven datasets analyzed (Fig. 1b and Supplementary Figure 1A). In addition, not only the mean expression but also the expression of the individual *MITF* isoforms were reduced in primary tumor  specimens when compared with the normal prostate tissue samples (Fig. 1c and Supplementary Figure 1B). Taken together, our data reveal MITF as a PGC1A-associated transcription factor that is consistently downregulated in PCa.

### MITF exhibits tumor-suppressive activity in PCa

**Fig. 2:**
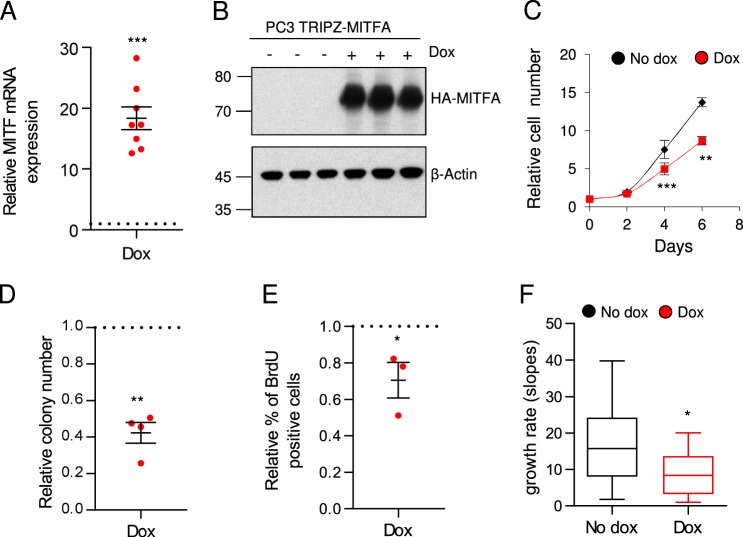
MITF exhibits tumor-suppressive activity in PC3 PCa cell line. **a**, **b** Analysis and quantification of MITF expression by qRTPCR (**a**, *n* = 8)) and western blot (**b**, representative experiment out of three independent ones) in PC3 TRIPZ-MITFA cells after treatment with 0.5 μg mL^−1^ doxycycline (Dox). **c** Relative cell number quantification by crystal violet in doxycycline-treated and nontreated PC3 TRIPZ-MITFA cells. Data are normalized to day 0. Asterisks indicate statistics of five independent experiments. One representative experiment out of five is shown. Error bars represent standard deviation. **d**, **e** Effect of MITF induction on anchorage-independent growth (**d**, soft agar; *n* = 4 independent experiments) and BrdU incorporation (**e**, *n* = 3 independent experiments). **f** Impact of MITF induction in tumor growth rate of PC3 TRIPZ-MITFA cells (*n* = 7 animals per group; 14 injections/tumors). No dox: MITFA noninduced conditions; Dox: MITFA-induced conditions. Error bars represent s.e.m. (**a**, **d**, and **e**) or minimum and maximum values (**f**). Statistic test: One-sample *t* test (**a**, **d**, and **e**) and Student’s *t* test (**c**, **f** *p < 0.05, **p < 0.01, ***p < 0.001

The expression profile of *MITF* in PCa, together with its direct correlation with *PGC1A*, was suggestive of a tumor-suppressive activity of the transcription factor. We first examined the differential expression of the distinct mRNA isoforms of *MITF* in normal, PCa primary tumors, and PCa cell lines (Supplementary Fig. 2A–C). *MITFA* was the isoform predominantly expressed in the three scenarios analyzed, and we pursued the studies further with this isoform. Next, we aimed to analyze the biological consequences of ectopic expression of MITFA in PC3 PCa cells. We transduced PC3 cells with a lentiviral vector containing a doxycycline-inducible cassette for the expression of MITFA resulting in the generation of the PC3 TRIPZ-MITFA cell line. The induction of MITFA expression (Fig. 2a, b) as well as the regulation of known target genes, including *PGC1A*^14,15^ (Supplementary Fig. 2 D–E) was confirmed. We next evaluated the biological outcome of MITFA ectopic expression in PC3 cells and observed that its upregulation significantly reduced two-dimensional and anchorage-independent growth (Fig. 2c, d), with no effect of doxycycline treatment by itself^14^. In line with its known function as an inhibitor of cell cycle progression^39^, the increased expression of MITFA in PC3 cells resulted in a decrease in BrdU incorporation, a surrogate readout of proliferation (Fig. 2e).

In order to ascertain whether the regulation of endogenous *PGC1A* (Supplementary Fig. 2E) was required for the antiproliferative effect of MITFA in PC3 cells, we aim at silencing *PGC1A* by using constitutive (pLKO) expression of short hairpins against it (Supplementary Fig. 2F). Transduction with the shRNA prevented the upregulation of *PGC1A* upon MITFA induction (Supplementary Fig. 2G) but the antiproliferative effect of the transcription factor remained unaffected (Supplementary Fig. 2H). These data suggested that the reduced proliferation induced by MITFA was not dependent on the regulation of endogenous *PGC1A* in PC3 cells.

Importantly, the overall reduction in cell proliferation induced by MITFA was confirmed in vivo. Using subcutaneous xenografts assays we observed that MITFA overexpression in PC3 cells (Supplementary Fig. 2I) led to a marked reduction in the tumor volume (Supplementary Fig. 2J) and growth rate (Fig. 2f), with no changes in angiogenesis (Supplementary Fig. 2K). Altogether these results demonstrate that MITFA isoform exhibits tumor-suppressive activity in PCa.

### Candidate screening of genes mediating the tumor-suppressive activity of MITF

**Fig. 3:**
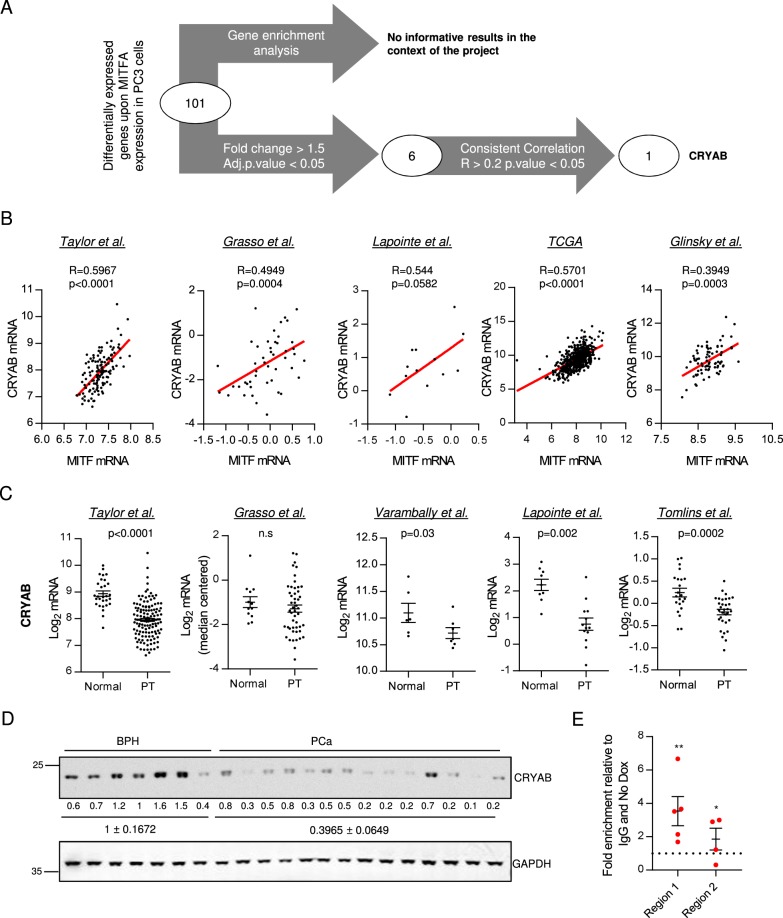
CRYAB is the candidate to mediate its tumor-suppressive activity in PCa. **a** Workflow of the candidate screening. **b** Correlation analysis between MITF and CRYAB expression in primary tumor (PT) specimens of different PCa datasets. Sample sizes: Taylor, *n* = 131; Grasso, *n* = 49; Lapointe, *n* = 13; TCGA provisional data, *n* = 495; and Glinsky, *n* = 78. **c** CRYAB expression in normal prostate and primary tumor (PT) specimens in different PCa datasets^9–13^. Sample sizes: Taylor (N, *n* = 29; PT, *n* = 130); Grasso (N, *n* = 12; PT, *n* = 49); Varambally (N, *n* = 6; PT, *n* = 7); Lapointe et al. (N, *n* = 9; PT, *n* = 13), and Tomlins (N, *n* = 22; PT, *n* = 32). Data from Taylor dataset correspond to the mean signal of all isoforms of the transcripts. In (**b**) and (**c**), each dot corresponds to an individual specimen. **d** Western blot analysis of CRYAB expression in benign prostatic hyperplasia (BPH) and PCa specimens from Basurto University Hospital cohort (BPH *n* = 7 patient specimens; PCa *n* = 14 patient specimens). **e** Chromatin immunoprecipitation (ChIP) of exogenous MITF on CRYAB promoter in PC3 TRIPZ-MITFA cells after induction with 0.5 µg mL^−1^ doxycycline for 3 days (*n* = 4–5). Binding to ANGPT4 was used as a negative control. Final data were normalized to IgG (negative-immunoprecipitation control) and to No dox condition. No dox: MITFA noninduced conditions; Dox: MITFA-induced conditions. Statistic tests: Spearman correlation (**b**); Mann−Whitney test (**c**); one-sample *t* test (**e**); Error bars represent s.e.m. *p < 0.05, **p < 0.01

In order to decipher the molecular mechanism driving the tumor-suppressive role of MITFA, we performed gene expression profiling of both doxycycline-treated and control PC3 TRIPZ-MITFA cells and identified 101 probes that showed statistically differential signal between both conditions (Supplementary Table 2; GEO Series accession number GSE114345). We first performed a gene enrichment analysis using the functional enrichment tool contained in CANCERTOOL^40^ with those genes that displayed upregulated expression (76 genes) upon MITFA overexpression (Fig. 3a and Supplementary Table 3), as the number of downregulated genes (25) was not sufficient to obtain any gene enrichment. Next, we aimed at identifying potential MITFA effectors of relevance in human PCa. To this end, we established a threshold of 1.5-fold change over MITFA noninduced cells, which resulted in eight probes (corresponding to six annotated genes) upregulated upon the induction of the transcription factor (Supplementary Table 2; yellow bold highlighted). We next performed correlation analysis between *MITF* and each of the six differentially expressed genes obtained from the microarray (Fig. 3a and Supplementary Figure 3A). The correlation analysis in PCa primary tumor specimens showed that a single gene, *Crystallin Alpha B (CRYAB)*, had a consistent correlation (in more than 50% of datasets) with *MITF*, both the mean of isoforms (Fig. 3b and Supplementary Figure 3A) and the individual isoform A (Supplementary Table 4). The *MITF−CRYAB* correlation was confirmed using an independent cohort of PCa patients from a local hospital (Basurto cohort, Supplementary Figure 3A). Moreover, the expression of CRYAB either at the level of mRNA (from public datasets and Basurto cohort) and protein (from Basurto cohort) was consistently downregulated through the progression of the disease (Fig. 3c, d and Supplementary Figure 3B–D), supporting the association of MITF and CRYAB expression in PCa.

The regulation of CRYAB expression by MITFA was further validated in vitro by western blot and quantitative real-time PCR (qRTPCR) in doxycycline-treated PC3 TRIPZ-MITFA cell lines and in vivo by qRTPCR in the xenograft samples (Supplementary Figure 3E–G). MITF is a transcription factor that regulates gene expression through the DNA binding to E-boxes (Myc-binding sites)^19^. In order to confirm the direct regulation of *CRYAB* expression by MITFA, we screened the promoter of the chaperon and performed chromatin immunoprecipitation assays in two Myc-binding sites (UCSC-Genome browser; Supplementary Figure 3H). As predicted, upon doxycycline treatment we detected differential binding of MITFA in both regions of *CRYAB* promoter (Fig. 3e).

Taken together, these data presented *CRYAB* as a direct target of MITFA and the best candidate to mediate its tumor-suppressive activity in PCa.

### CRYAB mediates the tumor-suppressive activity of MITF in PCa

**Fig. 4:**
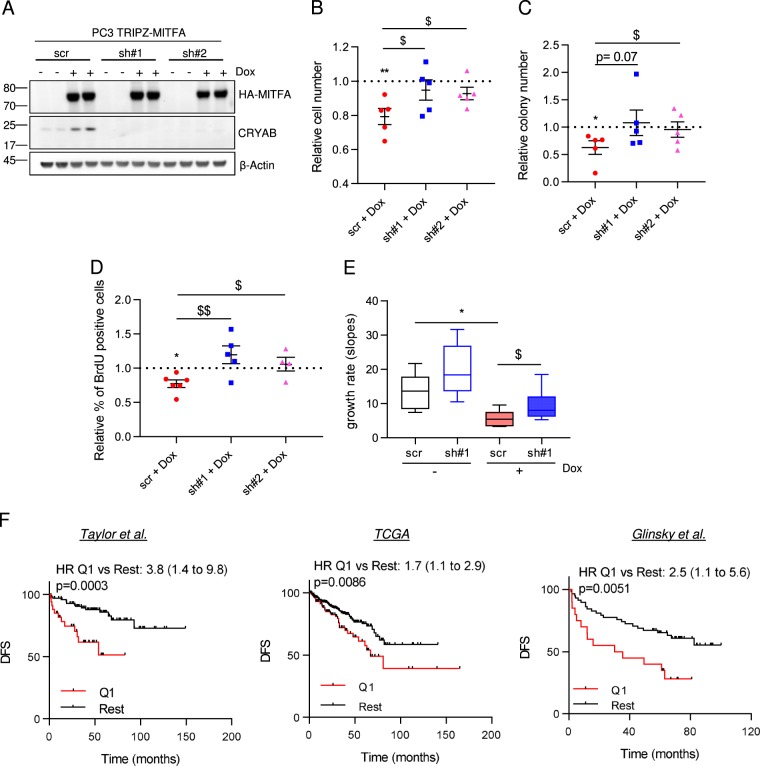
CRYAB mediates the tumor-suppressor activity of MITF. **a** Analysis of MITFA and CRYAB protein expression in doxycycline-treated PC3 TRIPZ-MITFA cells transduced with shScramble (scr) or two independent shCRYAB (sh#1 and#2) (one representative experiment with technical duplicates is shown; similar results were obtained in three independent experiments). **b** Relative cell number quantification by crystal violet in doxycycline-treated and nontreated PC3 TRIPZ-MITFA cells, in the presence (scr) or absence (sh#1, 2) of CRYAB (*n* = 5 independent experiments). Data are normalized to day 0 and represented as cell number at day 6 relative to No Dox condition (depicted by a dotted line). **c**, **d** Effect of CRYAB silencing on anchorage-independent growth (**c**, soft agar; *n* = 4 independent experiments) and BrdU incorporation (**d**, *n* = 3 independent experiments) in PC3 TRIPZ-MITFA cells after treatment with 0.5 μg mL^−1^ doxycycline. **e** Impact of CRYAB silencing on tumor growth rate of MITF-induced cells (*n* = 10 animals per group-scr or sh#1; 2 injections per mice (scr No dox, *n* = 10 tumors; sh#1 No dox, *n* = 8 tumors; scr Dox, *n* = 6 tumors; sh#1 Dox, *n* = 11 tumors). **f** Association of the mean signal of MITF and CRYAB with disease-free survival (DFS) in three PCa datasets (Q1: first quartile distribution; rest: second, third, and fourth quartile distribution. Sample sizes: Taylor, primary tumors *n* = 131; TCGA provisional data primary tumors *n* = 490; Glinsky, primary tumors *n* = 78. No dox: MITFA noninduced conditions; Dox: MITFA-induced conditions. HR: hazard ratio. Statistic tests: One-sample *t* test (**b**, **c** and **d**—No dox vs Dox conditions); Unpaired Student’s *t* test (*t*) (**b**, **c** and **d**—Dox-treated scr vs Dox-treated sh#1/2); Log-rank (Mantel–Cox) test (**f**). Error bars represent s.e.m. */$*p* < 0.05, **/$$*p* < 0.01. Asterisks indicate statistic between No dox and Dox conditions and dollar symbol between Dox-treated scr and Dox-treated sh#1 or 2

We next studied the functional relevance of CRYAB for the tumor-suppressive activity of MITFA in PCa. Towards this aim, we constitutively silenced the expression of *CRYAB* by RNAi using two independent short hairpin RNA (sh#1 and sh#2) in PC3 TRIPZ-MITFA cells. After validating that RNAi was achieved (Fig. 4a and Supplementary Figure 4A–C) the tumor-suppressive activity of MITFA was monitored in control and *CRYAB*-silenced conditions (PC3 TRIPZ-MITFA scr, sh#1 or sh#2 cell lines). *CRYAB* silencing blunted the antiproliferative effects of MITFA in vitro in two-dimensional and anchorage-independent growth when compared with scramble shRNA (Fig. 4b, c). Moreover, the reduction in BrdU induced by MITFA was prevented when *CRYAB* was silenced (Fig. 4d). Importantly, the requirement of CRYAB for the tumor-suppressive activity of MITFA was corroborated in vivo (Fig. 4e and Supplementary Figure 4D–F). The in vitro and in vivo data demonstrate that the induction of CRYAB is a major effector involved in the tumor-suppressive activity of the transcription factor MITF in PCa.

We next asked whether the functional association between MITF and CRYAB could be employed to identify PCa patients with high disease aggressiveness. We thus ascertained the stratification potential of the MITF-CRYAB axis in PCa by means of consistency and robustness. We download the mRNA expression raw data together with the clinical data (recurrence or not recurrence) from Taylor^11^, Glinsky^8^, and TCGA^7^ datasets. The individual or average expression signal of *CRYAB* and *MITF* genes was calculated for each patient in each dataset. Patients were separated by quartiles according to the individual or average signal of *CRYAB* and *MITF* genes and then Kaplan−Meyer survival curves were plotted comparing patients with low expression (Quartile 1—(Q1)) of the individual genes or the gene combination (*CRYAB* and *MITF)* vs the rest of the cohort (Q2 + Q3 + Q4). Strikingly, the signature formed by the average signal of *MITF* and *CRYAB* outperformed the prognostic potential of each individual gene, strongly suggesting that the pathway described herein is strongly associated to PCa aggressiveness (Fig. 4f and Supplementary Figure 5).

Our data provide solid evidence of an unprecedented MITF-CRYAB transcriptional axis that exerts tumor-suppressive activity in PCa and could positively contribute to disease prognosis.

## Discussion

Technological advances in the molecular understanding of cancer have led to a paradigmatic change in the way that we combat the disease^41^. We are now able to deconstruct a tumor at a molecular level using genomics, transcriptomics, proteomics, and metabolomics^42^. This, in turn, enables us to foresee, identify, and demonstrate the potential of patient stratification^3,14,43^. Specifically, the transcriptomics characterization of tumors is an invaluable strategy to identify clinically relevant genes that play key roles in the progression of cancer, especially for those types with poorer prognosis^14^. Thus, the comprehensive and integrative analysis of gene expression changes and clinical parameters in cancer has become a mainstream in cancer research^44–46^. Mining cancer-associated transcriptome datasets is an emerging approach used by top cancer research groups, but better tools are needed to increase its power and user-friendliness. In order to face this challenge, new interfaces to exploit OMICs data, such as cBioportal and CANCERTOOL^45,47,40^, are being designed to help scientists interrogate, integrate, and visualize large amount of information contained on multiple credible and qualified cancer datasets.

In the present study, we exploited publicly available and well-annotated (transcriptomics and clinical data) PCa databases together with experimental assays to describe a novel tumor-suppressive activity of the transcription factor MITF in PCa, which is executed, at least in part, through the direct regulation of CRYAB expression. The identification of MITF emanates from the screening of reported upstream regulators of PGC1A. It is worth noting that transcriptome-wide correlative study with the gene of interest could represent a complementary approach to predict candidate upstream regulators and downstream effectors.

The functional implication of MITF in cancer has been best defined in melanoma, in which the expression of the transcription factor is heterogeneous. Although some controversy exists regarding its oncogenic role in melanoma, MITF has been defined as a “lineage survival oncogene” with no data pointing out at a tumor-suppressive function^19,21,39,48–51^. Even though the expression of MITF has been detected in other cancer types^23,24,52^, no data supporting a functional role of MITF deregulation have been reported yet in a cancer scenario different from melanoma.

Here we show that MITF is downregulated in PCa when compared with normal specimens, in contrast to the elevated expression reported in hepatocellular carcinoma and chronic myeloid leukemia^23,52^. Moreover, the novelty of our study relies on the observation and definition of the tumor-suppressive activity of MITF in PCa. In this context, MITF upregulation was associated with a reduction in cell proliferation and DNA replication. As occurs in melanoma, the modulation of MITF expression in PCa cells induces the expression of the cell cycle inhibitor p21 but no changes in the cell cycle inhibitor p16 were observed (data not shown). Thus, our results in PCa are in line with the canonical function of MITF in cell cycle progression and proliferation in melanoma^39,50,51^.

It is important to highlight that the tissue-specific differences in MITF expression among different cancer types suggest that in order to fully comprehend MITF’s role in cancer, its expression and function has to be analyzed in the context of each particular cell and tissue type.

CRYAB is a member of the small heat-shock protein family that functions as stress-induced molecular chaperone. It inhibits the aggregation of denatured proteins, promotes cell survival, and inhibits apoptosis in the context of cancer^53–55^. Paradoxically, CRYAB is highly expressed in some cancer types but decreased in others and in both scenarios an association with cancer progression and prognosis has been reported^25,26,28–32,56–60^. In spite of the amount of information regarding the changes in CRYAB expression in cancer, the transcriptional regulation of this chaperone has been poorly explored^56^. In this study, we described a novel direct transcriptional regulation of CRYAB by MITF. Although there is no direct nor mechanistic evidence of the MITF-CRYAB transcriptional axis in other cancer types, in melanoma both MITF and CRYAB expression are upregulated by BRAF/MEK-inhibitor treatments^57,60^, suggesting that this regulation can go beyond both PCa scenario. Indeed, we observed that the correlation between MITF and CRYAB is also present in colorectal cancer, but not in breast nor lung cancer (data available in CANCERTOOL^40^).

In our study, the MITF-CRYAB transcriptional axis is reduced and exerts tumor-suppressive activity in PCa. This is in agreement with the reduced expression of CRYAB observed in PCa patients and its previous consideration as a protective gene against PCa^32^. Yet, the exact molecular mechanism underlying the tumor-suppressive activity of CRYAB remains to be elucidated.

Importantly, in the present manuscript, the extensive interrogation of PCa transcriptomes and associated clinical data has led us to propose the transcriptional axis MITF-CRYAB as a potential prognostic biomarker in PCa. The individual expression of CRYAB and MITF has been previously associated with poor prognosis in various tumor types^26,29–31,58,59^ and to therapy response in melanoma^61–63^. However, our data showing enhanced prognostic potential of the combined signature provides a new and exciting perspective of the functional interaction of these genes in PCa.

Our study endorses the potential of transcriptional deregulation analysis, as either a cause or a consequence of cancer, and its impact to support the discovery of novel cancer-related genes and long-term development of novel cancer treatment strategies.

## Materials and methods

### Cell culture and reagents

Human prostate carcinoma cell lines (PC3) were purchased from Leibniz-Institut DSMZ—Deutsche Sammlung von Mikroorganismen und Zellkulturen GmbH, who provided authentication certificate. The cell line used in this study was not found in the database of commonly misidentified cell lines maintained by ICLAC and NCBI Biosample. Cells were transduced with a modified TRIPZ (Dharmacon) doxycycline-inducible lentiviral construct in which the RFP and miR30 region was substituted by *HA-Flag-MITF*. Lentiviral shRNA constructs targeting *PGC1A* (#1-TRCN0000001165 and #2-TRCN0000001166) and *CRYAB* (#1-TRCN0000010822 and #2-TRCN0000010823) were purchased from Sigma and a scramble shRNA (hairpin sequence: CCGGCAACAAGATGAAGAGCACCAACTCGAGTTGGTGCTCTTCATCTTGTTG) was used as control. For *PGC1A* and *CRYAB* shRNAs, Puromycin resistance cassette was replaced by Hygromycin cassette from pLKO.1 Hygro (Addgene Ref. 24150) using *Bam*HI and *Kpn*I sites. Cell lines were routinely monitored for mycoplasma contamination and quarantined while treated if positive. Doxycycline hyclate (Dox) and Puromycin were purchased from Sigma, and Hygromycin from Invitrogen.

### Xenotransplant assays

All mouse experiments were carried out following the ethical guidelines established by the Biosafety and Welfare Committee at CIC bioGUNE. The procedures employed were carried out following the recommendations from AAALAC. Xenograft experiments were performed as previously described^14^, injecting 10^6^ cells per condition in two flanks per mouse (Nu/Nu immunodeficient males; 6–12 weeks of age). PC3 TRIPZ-HA-MITFA cells alone or under *CRYAB* silencing were injected in each flank of nude mice and 24 h post-injections mice were fed with chow or doxycycline diet (Research diets, D12100402).

### Patient samples

All samples were obtained from the Basque Biobank for research (BIOEF, Basurto University Hospital) upon informed consent and with evaluation and approval from the corresponding ethics committee (CEIC code OHEUN11-12 and OHEUN14-14).

### Molecular assays

Western blot was performed as previously described^14^. Antibodies used: HA-Tag (Cell Signalling #3724; dilution 1:10,000); MITF (Thermo Fisher Scientific MA5-14146; dilution 1:1000); β-Actin (Cell Signalling #3700; dilution 1:2000); GAPDH (clone 14C10; Cell Signalling #2218; dilution 1:1000); CRYAB (Cell Signalling #45844s; dilution 1:1000).

RNA was extracted using NucleoSpin® RNA isolation kit from Macherey-Nagel (ref: 740955.240C). For patients and animal tissues a Trizol-based implementation of the NucleoSpin® RNA isolation kit protocol was used as reported^14^. One microgram of total RNA was used for cDNA synthesis using Maxima^TM^ H Minus cDNA Synthesis Master Mix (Invitrogen M1682). Quantitative Real-Time PCR (qRTPCR) was performed as previously described^14^. Universal Probe Library (Roche) primers and probes employed are detailed in Supplementary Table 5. *GAPDH* (Hs02758991_g1) housekeeping assay from Applied Biosystems was used for data normalization.

For transcriptomics analysis in PC3 TRIPZ-HA-Flag-MITFA cells, Illumina whole genome -HumanHT-12_V4.0 (DirHyb, nt) method was used as reported^14^. A hypergeometric test was used to detect enriched dataset categories.

### Cellular assays

Cell number quantification with crystal violet was performed as referenced^14^.

For starvation experiments 100,000 cells per well were seeded in a six-well plate. Cells were initially plated in 10% FBS media for 24 h and then the media was changed to FBS free media and left overnight.

Soft agar assays were performed as previously described^14^, seeding 5000 cells per well in six-well plates.

For BrDu incorporation, cells were seeded on glass cover slips in 12-well plates and after 4 days, cells were incubated with 3 µg mL^−1^ BrDu (Sigma B5002). Cells were fixed with 4% para-formaldehyde, permeabilized with 1% Triton X-100 and incubated with a monoclonal anti-BrDu (MoBU-1) antibody (Invitrogen B35128) at a 1:100 dilution. Images were obtained with an AxioImager D1 microscope (Zeiss). At least three different areas per cover slip were quantified.

### Chromatin immunoprecipitation

Chromatin Immunoprecipitation (ChIP) was performed using the SimpleChIP^®^ Enzymatic Chromatin IP Kit (Cat: 9003, Cell Signalling Technology, Inc). Four million PC3 cells were grown in 150 mm dishes either with or without 0.5 µg mL^−1^ doxycycline during 3 days. Cells from three 150 mm dishes were cross-linked with 35% formaldehyde for 10 min at room temperature. Glycine was added to dishes during 5 min at room temperature. Cells were then washed twice with ice-cold PBS, and scraped into PBS + PMSF. Pelleted cells were lysed and nuclei were harvested following the manufacturer’s instructions. Nuclear lysates were digested with micrococcal nuclease for 20 min at 37 °C and then sonicated in 500 μL aliquots on ice for 6 pulses of 20 s using a Branson sonicator. Cells were held on ice for at least 1 min between sonications. Lysates were clarified at 11,000 × *g* for 10 min at 4 °C, and chromatin was stored at −80 °C. HA-Tag polyclonal antibody (Cat: C29F4, Cell Signalling Technology) and IgG antibody (Cat: 2729, Cell Signalling Technology, Inc), were incubated overnight (4 °C) with rotation and protein G magnetic beads were incubated 2 h (4 °C). Washes and elution of chromatin were performed following the manufacturer’s instructions. DNA quantification was carried out using a Viia7 Real-Time PCR System (Applied Biosystems) with SybrGreen reagents and primers that amplify the predicted MITFA binding region to *CRYAB* (region 1; For: ttgtttcctcgtagggcttg, Rev: tttcagagccaggagagagc- region 2; For: tctggaatggtgatgtcagg, Rev: attgggtgtggacagaaagc) and *ANGPTL4* (For: gttgacccggctcacaat, Rev: ggaacagctcctggcaatc) as a negative binding control.

### Whole-genome gene expression characterization

Whole-genome expression characterization was conducted using Human HT12 v4 BeadChips (Illumina Inc.). In brief, cRNA synthesis was obtained with TargetAmp™ Nano-g™ Biotin-aRNA Labeling Kit for the Illumina® System, Epicentre (Cat. Num. TAN07924) and subsequent amplification, labeling and hybridization were performed according to Whole-Genome Gene Expression Direct Hybridization Illumina Inc.’s protocol. Raw data were extracted with GenomeStudio analysis software (Illumina Inc.) in the form of GenomeStudio’s Final Report (sample probe profile).

### Bioinformatics analysis and statistics

#### Database normalization

All the datasets used for the data mining analysis were downloaded from GEO and TCGA. Referenced accessions: TCGA https://cancergenome.nih.gov/, Grasso et al., GEO: GSE35988 (9); Lapointe et al., GEO: GSE3933 (10); Taylor et al., GEO: GSE21032 (11); Tomlins et al., GEO: GSE6099 (12); Varambally et al., GEO: GSE3325 (13); and Glinsky et al (8). GEO-downloaded data were subjected to background correction, log_2_ transformation and quartile normalization. In the case of using a preprocessed dataset, this normalization was reviewed and corrected if required. TCGA data were downloaded as upper quartile normalized RSEM count, which was been log2 transformed.

#### Quartile analysis in disease-free survival

Patients biopsies from primary tumors were organized into four quartiles according to the expression of the gene of interest in three datasets. The recurrence of the disease was selected as the event of interest. Kaplan−Meier estimator was used to perform the test as it takes into account *right-censoring*, which occurs if a patient withdraws from a study.

#### Correlation analysis

Spearman correlation test was applied to analyze the relationship between paired genes.

#### Gene enrichment

The recently published tool, CANCERTOOL^40^, harbors 11 independent enrichment databases, including the basic Gene Ontology analysis (GO, biological process (GOBP), molecular function (GOMF) and cell compartment (GOCC)), pathways and pathophysiological processes (KEGG, Biocarta, Reactome, Biocarta, Onco, DOSE, HIPC, Connectivity Map), and the upstream regulatory cue prediction tool (TFT, MIR). The prevalence of such functions within the gene list was analyzed, and statistical significance of the associations sieved according to the Benjamini–Hochberg correction (adjusted *p* value).

#### Statistical analysis

No statistical method was used to predetermine the sample size. The experiments were not randomized. The investigators were not blinded to allocation during experiments and outcome assessment. Unless otherwise stated, data analyzed by parametric tests are represented by the mean ± s.e.m. of pooled experiments and median ± interquartile range for experiments analyzed by nonparametric tests. *n* values represent the number of independent experiments performed, the number of individual mice or patient specimens. For each independent in vitro experiment, at least two technical replicates were used and a minimum number of three experiments were done to ensure adequate statistical power. For data mining analysis Student’s *t* test for two component comparisons. In the in vitro experiments, normal distribution was confirmed or assumed (for *n* < 5) and Student’s *t* test was applied for two component comparisons. In the statistical analyses involving fold changes, one-sample *t* test with a hypothetical value of 1 was performed. The confidence level used for all the statistical analyses was of 95% (alpha value = 0.05). Two-tail statistical analysis was applied for experimental design without predicted result, and one-tail for validation or hypothesis-driven experiments.

#### Gene expression array data analysis

First, raw expression data were background-corrected, log2-transformed, and quantile-normalized using the lumi R package^64^, available through the Bioconductor repository^65,66^. Probes with a “detection *p* value” lower than 0.01 in at least one sample were considered expressed. For the detection of differentially expressed genes, a linear model was fitted to the probe data and empirical Bayes moderated *t* statistics were calculated using the limma^67^ package from Bioconductor. Adjusted *p* values were estimated with Benjamini−Hochberg false discovery rate method (https://www.jstor.org/stable/2346101)^68^. Only genes with differential fold-change (FC) > 1.5 or <−1.5 and an adjusted *p* value < 0.05 were considered as differentially expressed. The transcriptomics data generated in this publication have been deposited in NCBI’s Gene Expression Omnibus and are accessible through GEO Series accession number GSE114345.

## Electronic supplementary material

Supplementary figure legends

Supplementary figure 1

Supplementary figure 2

Supplementary figure 3

Supplementary figure 4

Supplementary figure 5

Supplementary Table 1

Supplementary table 2

Supplementary Table 3

Supplementary Table 4

Supplementary Table 5

## References

[1] Hanahan D, Weinberg RA (2011). Hallmarks of cancer: the next generation. Cell.

[2] Martin-Martin N, Carracedo A, Torrano V (2017). Metabolism and transcription in cancer: Merging Two Classic Tales. Front. Cell. Dev. Biol..

[3] Martin-Martin N (2016). Stratification and therapeutic potential of PML in metastatic breast cancer. Nat. Commun..

[4] Martin-Martin N (2018). PPARdelta elicits ligand-independent repression of trefoil factor family to limit prostate cancer growth. Cancer Res..

[5] Bacolod MD (2015). Examination of epigenetic and other molecular factors associated with mda-9/syntenin dysregulation in cancer through integrated analyses of public genomic datasets. Adv. Cancer Res..

[6] Olvedy M (2017). Comparative oncogenomics identifies tyrosine kinase FES as a tumor suppressor in melanoma. J. Clin. Invest..

[7] Cancer Genome Atlas Research N. (2015). The molecular taxonomy of primary prostate. Cancer Cell..

[8] Glinsky GV, Glinskii AB, Stephenson AJ, Hoffman RM, Gerald WL (2004). Gene expression profiling predicts clinical outcome of prostate cancer. J. Clin. Invest..

[9] Grasso CS (2012). The mutational landscape of lethal castration-resistant prostate cancer. Nature.

[10] Lapointe J (2007). Genomic profiling reveals alternative genetic pathways of prostate tumorigenesis. Cancer Res..

[11] Taylor BS (2010). Integrative genomic profiling of human prostate cancer. Cancer Cell..

[12] Tomlins SA (2007). Integrative molecular concept modeling of prostate cancer progression. Nat. Genet..

[13] Varambally S (2005). Integrative genomic and proteomic analysis of prostate cancer reveals signatures of metastatic progression. Cancer Cell.

[14] Torrano V (2016). The metabolic co-regulator PGC1alpha suppresses prostate cancer metastasis. Nat. Cell Biol..

[15] Valcarcel-Jimenez L, Torrano V, Carracedo A (2017). New insights on prostate cancer progression. Cell Cycle.

[16] Valcarcel-Jimenez L, Gaude E, Torrano V, Frezza C, Carracedo A (2017). Mitochondrial metabolism: yin and yang for tumor progression. Trends Endocrinol. Metab..

[17] Hock MB, Kralli A (2009). Transcriptional control of mitochondrial biogenesis and function. Annu. Rev. Physiol..

[18] Haq R (2013). Oncogenic BRAF regulates oxidative metabolism via PGC1alpha and MITF. Cancer Cell.

[19] Wellbrock C, Arozarena I (2015). Microphthalmia-associated transcription factor in melanoma development and MAP-kinase pathway targeted therapy. Pigment. Cell. Melanoma Res..

[20] Tachibana M (2000). MITF: a stream flowing for pigment cells. Pigment Cell Res..

[21] Garraway LA (2005). Integrative genomic analyses identify MITF as a lineage survival oncogene amplified in malignant melanoma. Nature.

[22] Vazquez F (2013). PGC1alpha expression defines a subset of human melanoma tumors with increased mitochondrial capacity and resistance to oxidative stress. Cancer Cell..

[23] Aggoune D (2017). Bone marrow mesenchymal stromal cell (MSC) gene profiling in chronic myeloid leukemia (CML) patients at diagnosis and in deep molecular response induced by tyrosine kinase inhibitors (TKIs). Leuk. Res..

[24] Li Y, Kong D, Ahmad A, Bao B, Sarkar FH (2012). Targeting bone remodeling by isoflavone and 3,3’-diindolylmethane in the context of prostate cancer bone metastasis. PLoS ONE.

[25] Moyano JV (2006). AlphaB-crystallin is a novel oncoprotein that predicts poor clinical outcome in breast cancer. J. Clin. Invest..

[26] Voduc, K. D. et al. alphaB-crystallin expression in breast cancer is associated with brain metastasis. *NPJ Breast Cancer*, **1** (2015). 10.1038/npjbcancer.2015.14 PMC5027912 27656679

[27] Shi C, Yang X, Bu X, Hou N, Chen P (2017). Alpha B-crystallin promotes the invasion and metastasis of colorectal cancer via epithelial-mesenchymal transition. Biochem. Biophys. Res. Commun..

[28] Yilmaz M (2015). Alpha-B-crystallin expression in human laryngeal squamous cell carcinoma tissues. Head. Neck.

[29] Volkmann J (2013). High expression of crystallin alphaB represents an independent molecular marker for unfavourable ovarian cancer patient outcome and impairs TRAIL- and cisplatin-induced apoptosis in human ovarian cancer cells. Int. J. Cancer.

[30] Qin H (2014). Elevated expression of CRYAB predicts unfavorable prognosis in non-small cell lung cancer. Med. Oncol..

[31] Shi C (2014). Alpha B-crystallin correlates with poor survival in colorectal cancer. Int. J. Clin. Exp. Pathol..

[32] Altintas DM (2013). Differentially expressed androgen-regulated genes in androgen-sensitive tissues reveal potential biomarkers of early prostate cancer. PLoS ONE.

[33] Huang Z (2012). Tumor suppressor Alpha B-crystallin (CRYAB) associates with the cadherin/catenin adherens junction and impairs NPC progression-associated properties. Oncogene.

[34] Borniquel S (2010). Inactivation of Foxo3a and subsequent downregulation of PGC-1 alpha mediate nitric oxide-induced endothelial cell migration. Mol. Cell. Biol..

[35] Jin J (2013). Transcriptional and translational regulation of C/EBPbeta-HDAC1 protein complexes controls different levels ofp53, SIRT1, and PGC1alpha proteins at the early and late stages of liver cancer. J. Biol. Chem..

[36] Sancho P (2015). MYC/PGC-1alpha balance determines the metabolic phenotype and plasticity of pancreatic cancer stem cells. Cell Metab..

[37] Shimizu YI (2009). Fasting induced up-regulation of activating transcription factor 5 in mouse liver. Life. Sci..

[38] Wende AR (2015). Enhanced cardiac Akt/protein kinase B signaling contributes to pathological cardiac hypertrophy in part by impairing mitochondrial function via transcriptional repression of mitochondrion-targeted nuclear genes. Mol. Cell. Biol..

[39] Carreira S (2005). Mitf cooperates with Rb1 and activates p21Cip1 expression to regulate cell cycle progression. Nature.

[40] Cortazar, A. R. et al. CANCERTOOL, a visualization and representation interface to exploit cancer datasets. *Cancer Res.* (2018). 10.1158/0008-5472.CAN-18-1669 30232219

[41] Wang G, Zhao D, Spring DJ, DePinho RA (2018). Genetics and biology of prostate cancer. Genes Dev..

[42] Karczewski KJ, Snyder MP (2018). Integrative omics for health and disease. Nat. Rev. Genet..

[43] Carracedo A (2012). A metabolic prosurvival role for PML in breast cancer. J. Clin. Invest..

[44] Cheng PF, Dummer R, Levesque MP (2015). Data mining: The Cancer Genome Atlas in the era of precision cancer medicine. Swiss Med. Wkly..

[45] Gao J (2013). Integrative analysis of complex cancer genomics and clinical profiles using the cBioPortal. Sci. Signal..

[46] Klonowska K (2016). Oncogenomic portals for the visualization and analysis of genome-wide cancer data. Oncotarget.

[47] Cerami E (2012). The cBio cancer genomics portal: an open platform for exploring multidimensional cancer genomics data. Cancer Discov..

[48] Carreira S (2006). Mitf regulation of Dia1 controls melanoma proliferation and invasiveness. Genes Dev..

[49] Vachtenheim J, Ondrusova L (2015). Microphthalmia-associated transcription factor expression levels in melanoma cells contribute to cell invasion and proliferation. Exp. Dermatol..

[50] Wellbrock C, Marais R (2005). Elevated expression of MITF counteracts B-RAF-stimulated melanocyte and melanoma cell proliferation. J. Cell. Biol..

[51] Wellbrock C (2008). Oncogenic BRAF regulates melanoma proliferation through the lineage specific factor MITF. PLoS ONE.

[52] Thomaschewski M (2017). Multi-color RGB marking enables clonality assessment of liver tumors in a murine xenograft model. Oncotarget.

[53] Kamradt MC (2005). The small heat shock protein alpha B-crystallin is a novel inhibitor of TRAIL-induced apoptosis that suppresses the activation of caspase-3. J. Biol. Chem..

[54] Clark JI, Muchowski PJ (2000). Small heat-shock proteins and their potential role in human disease. Curr. Opin. Struct. Biol..

[55] Goplen D (2010). alphaB-crystallin is elevated in highly infiltrative apoptosis-resistant glioblastoma cells. Am. J. Pathol..

[56] Zhang L (2016). Kruppel-like factor 4 promotes human osteosarcoma growth and metastasis via regulating CRYAB expression. Oncotarget.

[57] Hu R, Aplin AE (2010). alphaB-crystallin is mutant B-RAF regulated and contributes to cyclin D1 turnover in melanocytic cells. Pigment. Cell. Melanoma Res..

[58] Chin D (2005). Alpha B-crystallin, a new independent marker for poor prognosis in head and neck cancer. Laryngoscope.

[59] Shi QM (2016). High level of alphaB-crystallin contributes to the progression of osteosarcoma. Oncotarget.

[60] Smith MP (2016). Inhibiting drivers of non-mutational drug tolerance is a salvage strategy for targeted melanoma therapy. Cancer Cell.

[61] Muller J (2014). Low MITF/AXL ratio predicts early resistance to multiple targeted drugs in melanoma. Nat. Commun..

[62] Naffouje S, Naffouje R, Bhagwandin S, Salti GI (2015). Microphthalmia transcription factor in malignant melanoma predicts occult sentinel lymph node metastases and survival. Melanoma Res..

[63] Najem A (2017). P53 and MITF/Bcl-2 identified as key pathways in the acquired resistance of NRAS-mutant melanoma to MEK inhibition. Eur. J. Cancer.

[64] Du P, Kibbe WA, Lin SM (2008). lumi: a pipeline for processing Illumina microarray. Bioinformatics.

[65] Gentleman RC (2004). Bioconductor: open software development for computational biology and bioinformatics. Genome Biol..

[66] Huber W (2015). Orchestrating high-throughput genomic analysis with Bioconductor. Nat. Methods.

[67] Ritchie ME (2015). limma powers differential expression analyses for RNA-sequencing and microarray studies. Nucleic Acids Res..

[68] Benjamini Y, Hochberg Y (1995). Controlling the false discovery rate: a practical and powerful approach to multiple testing. J. R. Stat. Soc. Ser. B (Methodol.).

