# Supplementary material for: Integrative analysis of transcriptomics and clinical data uncovers the tumor-suppressive activity of MITF in prostate cancer
Source: Cell Death Dis. 2018 Oct 11;9(10):1041. doi: 10.1038/s41419-018-1096-6 (PMC6181952; doi:10.1038/s41419-018-1096-6)
Supplement: Supplementary file 1 — Supplementary figure legends [file 41419_2018_1096_MOESM1_ESM.docx]

**Supplementary figure legends**

**Supplementary Figure 1. A.** Heat-maps showing the correlation of PGC1A expression with a panel of transcription factors that regulates its expression. NA: not applicable. **B.** Gene expression analysis of the different MITF isoforms in normal and primary tumors of Taylor data set. Each dot corresponds to an individual specimen. Sample size (normal, n=29; primary tumor (PT), n=131). Statistic test: Spearman correlation *R* (A) and Mann Whitney test (B). p, p-value.

**Supplementary Figure 2. A-C**. Gene expression analysis of the different MITF isoforms in (A) normal, (B) primary tumors and (C) cell lines of Taylor dataset (11). Each Log2 mRNA value corresponding to an individual specimen (dots) was converted into antilog and normalized to MITFA isoform. Sample size (normal, n=29; primary tumor, n=131; cell lines, n=19). **D.** Analysis of PGC1A expression by qRTPCR in PC3 TRIPZ-MITFA cells (D, n=8 independent experiments) upon doxycycline treatment. Expression data is normalized to No dox condition, depicted by a dotted line. **E**. Schematic representation of the experimental approach to silence PGC1A in non-treated and Dox-treated PC3 TRIPZ-MITFA cells. **F**. Confirmation of PGC1A silencing by qRTPCR in PC3 TRIPZ-MITFA cells transduced with shScramble (scr) or shPGC1A (sh#1 and #2) (n=4 independent experiments) and treated with doxycycline. Expression data is normalized to scr No dox condition, depicted by a dotted line. **G.** Relative cell number quantification by crystal violet of PC3 TRIPZ-MITFA scr or shPGC1A non-treated and doxycycline-treated cells (n=4 independent experiments). Data is normalized to day 0 and represented as cell number at day 4 relative to each No dox condition, depicted by a dotted line. **H**. Validation of MITF induction by qRTPCR in xenograft samples (n=13 No dox; n=11 Dox; tumor samples). **I**. Evaluation of tumor growth in xenotransplantation experiments. 10^6^ PC3 TRIPZ-MITFA cells were injected in each flank of nude mice and 24hr post-injections mice were fed with chow or doxycycline diet (n=7 animals; 14 injections/tumors). Tumor volume from each individual injection was monitored for 25 days. Error bars represent s.e.m (A, B, C, D, F, G and H) or minimun and maximum values (I). Statistic test: ANOVA (A, B and C), one-sample *t*-test (D, F and G- No dox vs Dox conditions), Mann Whitney U test (H and I), unpaired Student *t*-test (F and G- Dox-treated scr vs Dox-treated sh#1/2). No dox: MITFA non-induced conditions; Dox: MITFA induced conditions. p, p-value. ***p < 0.001, **p < 0.01, *p < 0.05. Asterisks indicate statistic between No dox and Dox conditions and dollar symbol between scr and sh#1 or 2.

**Supplementary Figure 3**. **A.** Heat-map showing correlations between MITF and the panel of differentially expressed genes upon MITF induction in PC3 TRIPZ-MITFA cells. **B**. Correlation analysis between MITF and CRYAB expression in PCa specimens (n=16) from Basurto University Hospital cohort. Each dot corresponds to an individual specimen. **C**. qRTPCR mRNA expression analysis of CRYAB in benign prostatic hyperplasia (BPH) and PCa specimens from Basurto University Hospital cohort (BPH n=6 patient specimens; PCa n=16 patient specimens) **D.** CRYAB protein expression quantification in benign prostatic hyperplasia (BPH) and PCa specimens from Basurto University Hospital cohort (BPH n=7 patient specimens; PCa n=14 patient specimens). Data from B and C is normalized to BHP specimens, depicted by a dotted line. **E-F**. *In vitro* validation of CRYAB upregulation by protein (D, n=3 independent experiments with technical duplicates are shown) and qRTPCR mRNA (E, n=9 independent experiments) expression analyses in non-treated and doxycycline-treated PC3 TRIPZ-MITFA cells. Data in E is normalized to No dox condition, depicted by a dotted line. **G.** *In vivo* validation of CRYAB upregulation by qRTPCR in xenograft samples of PC3 TRIPZ-MITFA cells (F, n=12 both No dox and Dox samples). Data is normalized to No dox condition, depicted by a dotted line in E. **H**. Panel representing the different regions (R1-R2) chosen for the analysis of MITFA occupancy on CRYAB promoter in PC3 TRIPZ-MITFA cells. No dox: MITFA non-induced conditions; Dox: MITFA induced conditions. p, p-value. **p < 0.01. Statistic test: Spearman correlation (A); One-sample test (E); Mann Whitney test (B, D and F).

**Supplementary Figure 4**. **A.** Schematic representation of the experimental approach to silence CRYAB expression in PC3 TRIPZ-MITFA cells using constitutive shRNA. **B and C.** Analysis of MITF (B) and CRYAB (C) (n=5 independent experiments) expression by qRTPCR in non-treated and doxycycline-treated PC3 TRIPZ-MITFA cells transduced with either scramble (scr) or CRYAB shRNA (sh#1/2). Data is normalized to untreated scr condition. **D and E**. Analysis of MITF (D) and CRYAB (E) expression by qRTPCR in xenograft samples of PC3 TRIPZ-MITFA cells (scr No dox, n=10 tumors; sh#1 No dox, n=8 tumors; scr Dox, n=6 tumors; sh#1 Dox, n=11 tumors). Data is normalized to untreated scr condition. **F**. Evaluation of tumor growth in xenotransplantation experiments. 10^6^ PC3 TRIPZ-MITFA cells transduced with scr or sh#1 were injected in each flank of nude mice and 24hr post-injections mice were fed with chow or doxycycline diet (n=10 animals per group-scr or sh#1; 2 injections per mice; (scr No dox, n=10 tumors; sh#1 No dox, n=8 tumors; scr Dox, n=6 tumors; sh#1 Dox, n=11 tumors). Tumor volume was monitored during 21 days. No dox: MITFA non-induced conditions; Dox: MITFA induced conditions. sh#1&2: two independent CRYAB short hairpin (sh) RNAs. Statistic tests: One sample t test (B and C); Student t-test (B and C- between conditions different from untreated scr; D and E); Wilcoxon test (F). */$ p < 0.05, **/$$ p < 0.01, ***/$$$ p < 0.001. Asterisks indicate statistic between No dox and Dox conditions in each cell line or between scr No dox and the rest of the conditions; dollar symbol indicate statistic between dox-treated scr and dox-treated sh#1 or 2.

**Supplementary Figure 5.** Association of the MITF and CRYAB expression with disease-free survival (DFS) in three PCa data sets (Q1: first quartile distribution; rest: sum of second, third and fourth quartile distribution). Sample sizes: Taylor, primary tumors n=131; TCGA provisional data primary tumors n=490; Glinsky, primary tumors n=78.
