# Supplementary figures and images for: Integrative analysis of transcriptomics and clinical data uncovers the tumor-suppressive activity of MITF in prostate cancer
Source: Cell Death Dis. 2018 Oct 11;9(10):1041. doi: 10.1038/s41419-018-1096-6 (PMC6181952; doi:10.1038/s41419-018-1096-6)

A

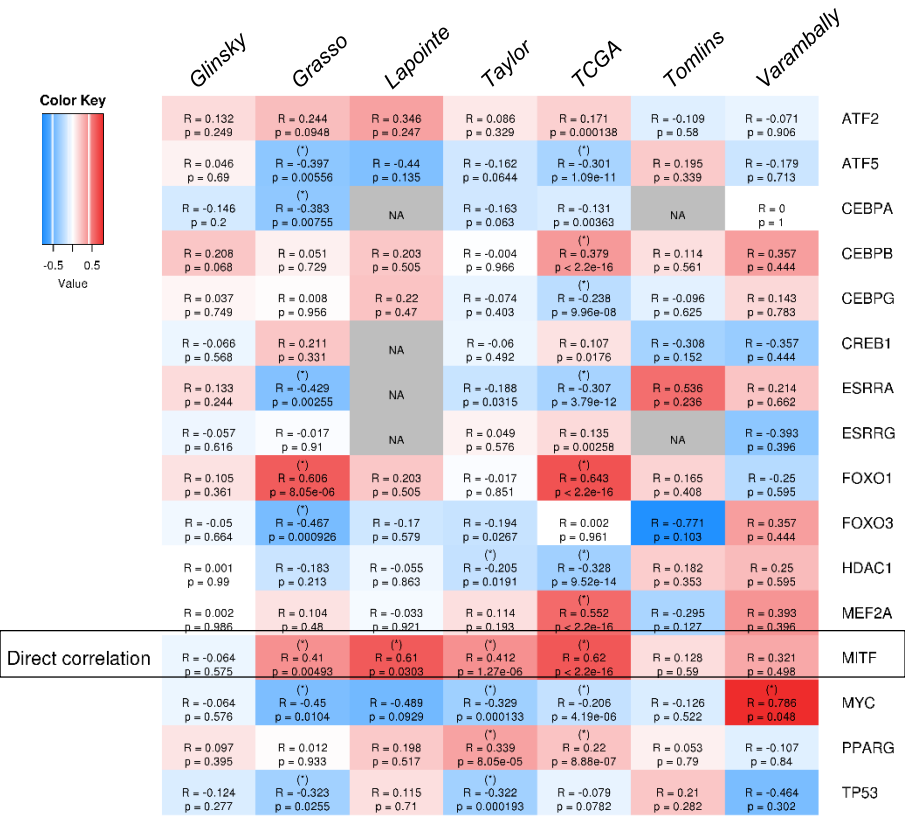

B

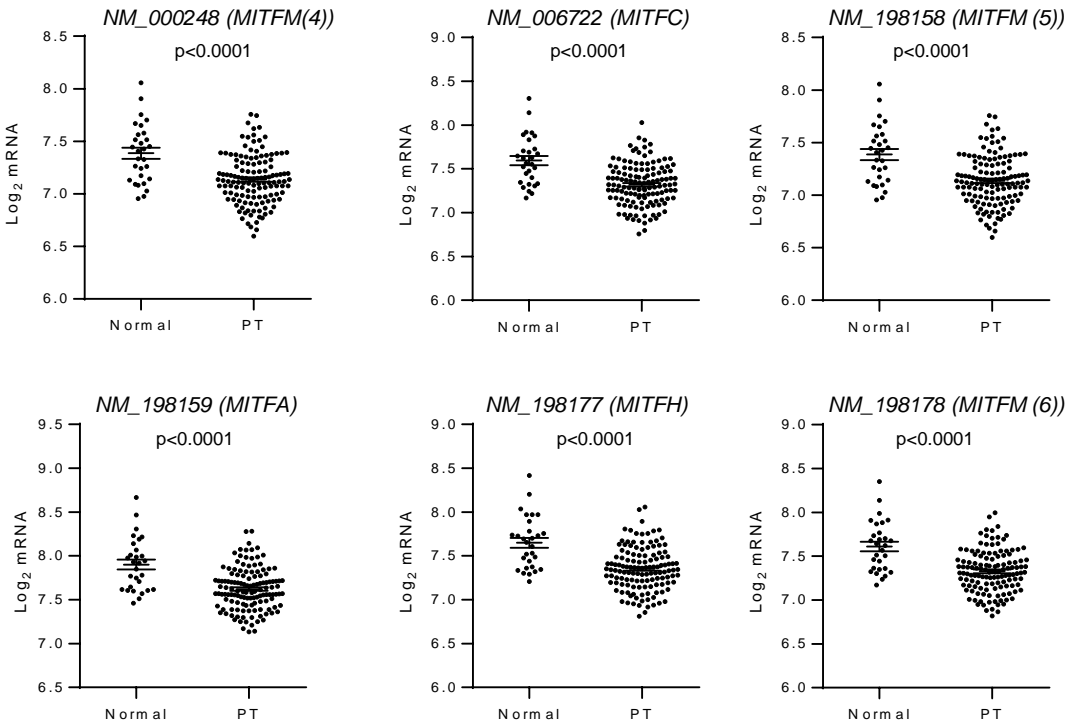

Supplement: Supplementary file 2 — Supplementary figure 1 [file 41419_2018_1096_MOESM2_ESM.pdf]

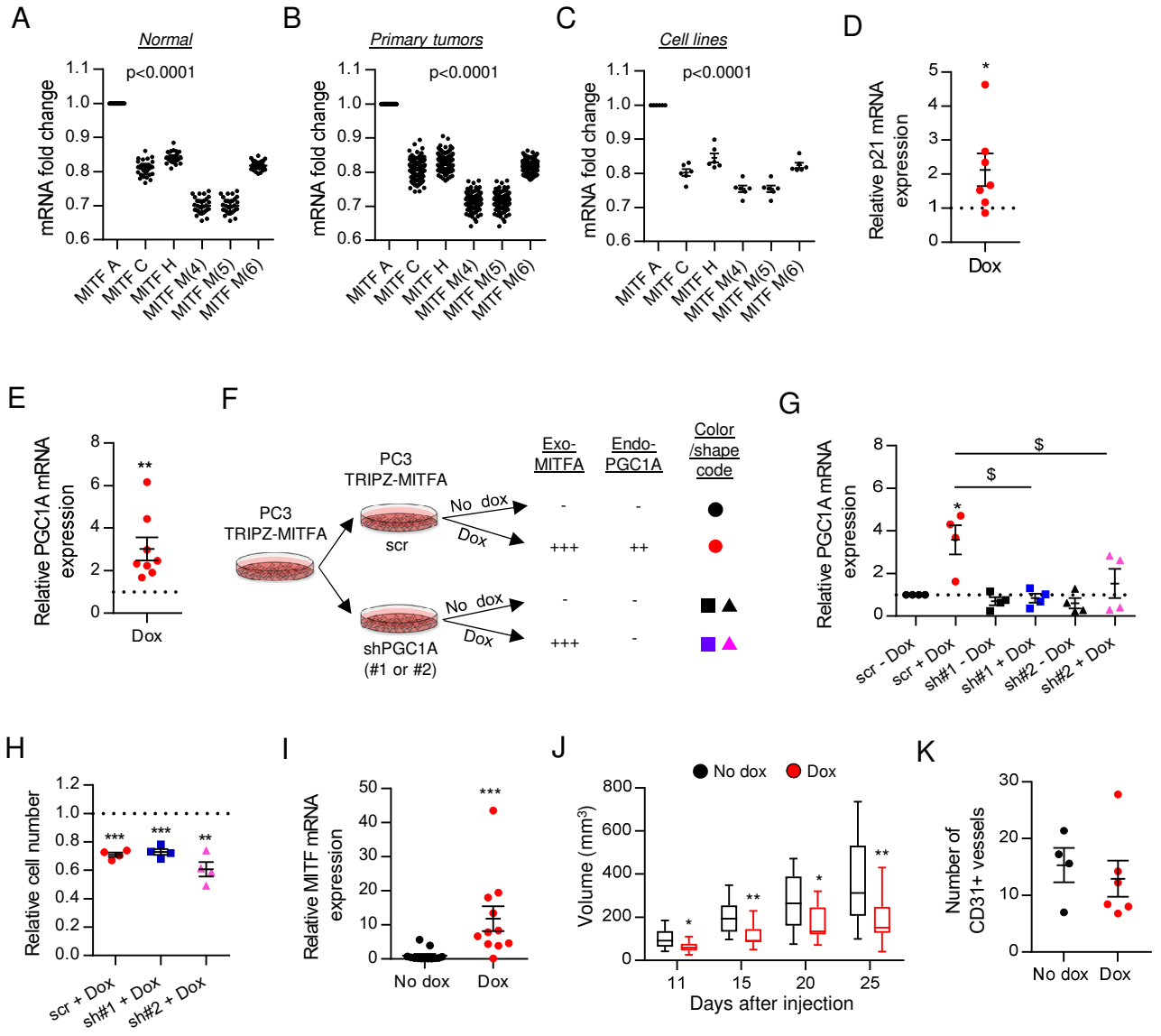

Supplement: Supplementary file 3 — Supplementary figure 2 [file 41419_2018_1096_MOESM3_ESM.pdf]

A

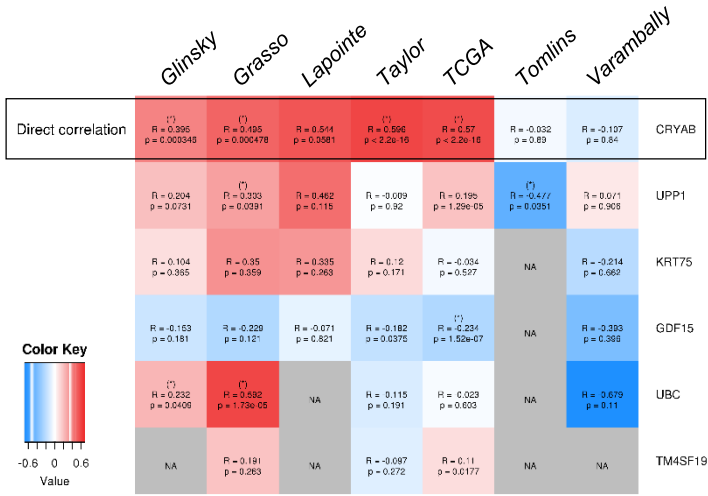

B

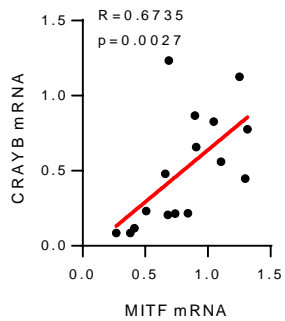

C

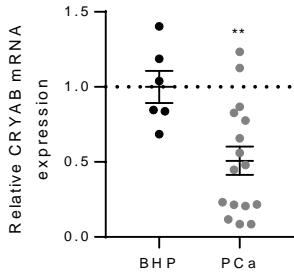

D

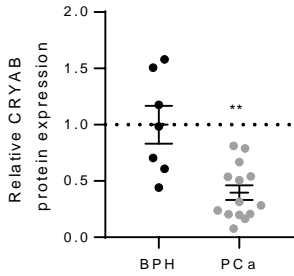

E

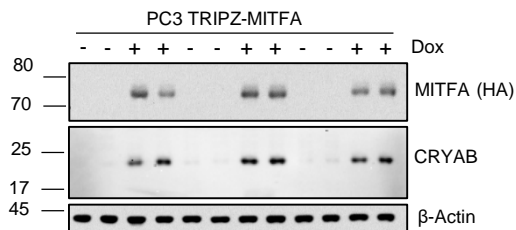

F

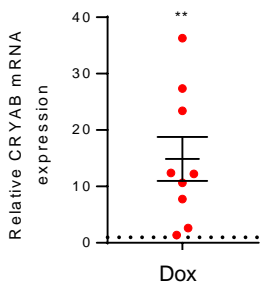

G

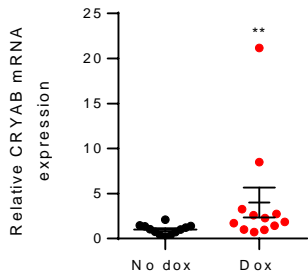

H

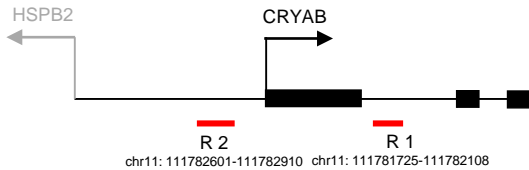

Supplement: Supplementary file 4 — Supplementary figure 3 [file 41419_2018_1096_MOESM4_ESM.pdf]

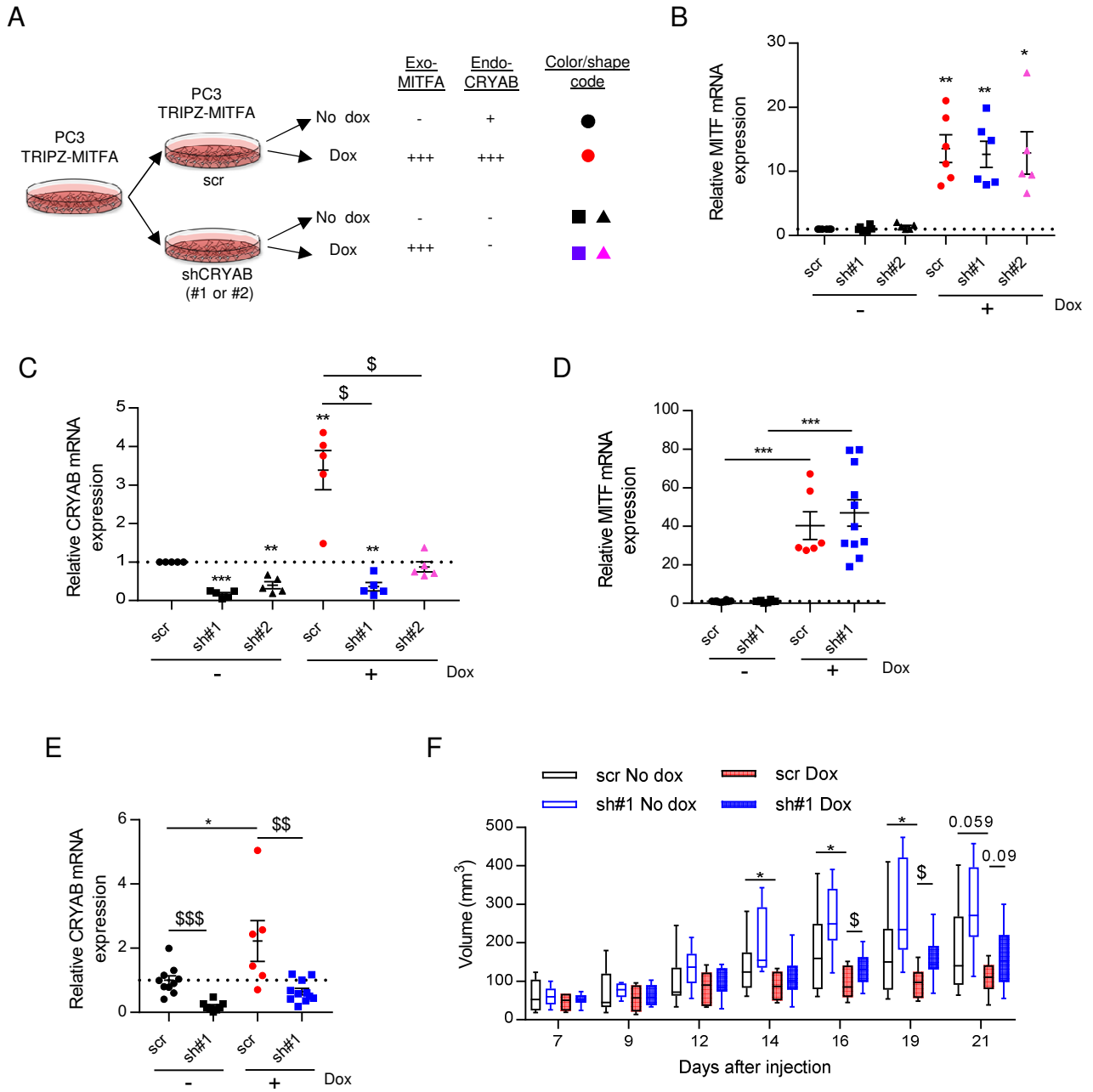

Supplement: Supplementary file 5 — Supplementary figure 4 [file 41419_2018_1096_MOESM5_ESM.pdf]

*Taylor et al.*

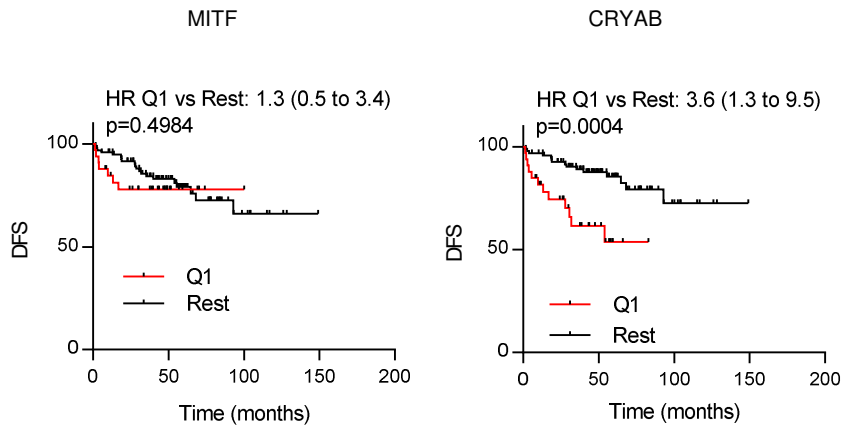

*TCGA*

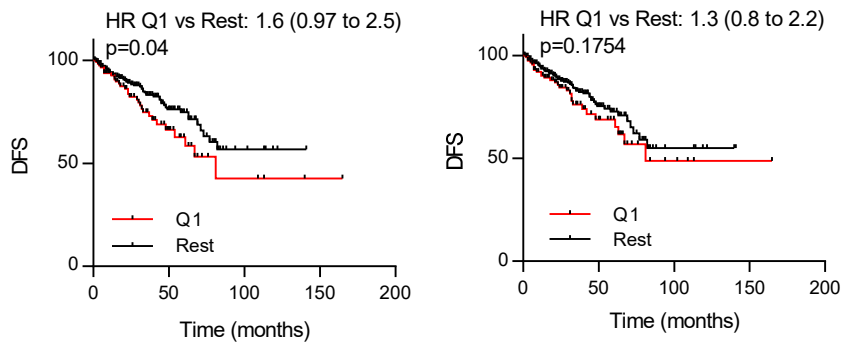

*Glinsky et al.*

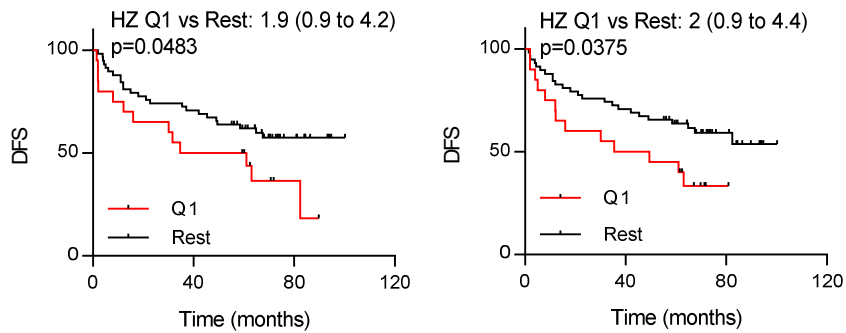

Supplement: Supplementary file 6 — Supplementary figure 5 [file 41419_2018_1096_MOESM6_ESM.pdf]
