# Supplementary material for: Integrative analysis of transcriptomics and clinical data uncovers the tumor-suppressive activity of MITF in prostate cancer
Source: Cell Death Dis. 2018 Oct 11;9(10):1041. doi: 10.1038/s41419-018-1096-6 (PMC6181952; doi:10.1038/s41419-018-1096-6)
Supplement: Supplementary file 8 — Supplementary table 2 [file 41419_2018_1096_MOESM8_ESM.pdf]

regul	ProbeID	SYMBOL	Acc_No	FoldChange	LogRatio	PValue	AdjPValue	mean_g2	mean_g1	Probe_Seque	Gene_descr
UU	ILMN_180832	TM4SF19	XM_0011342	2,3615	1,2397	6,48E-16	1,25E-11	10,52989	9,29019	GCCCTTCTGT	PREDICTED: Homo sapiens transmembrane 4 L six family member 19, transcript variant 2 (TM4SF19), mRNA.
UU	ILMN_241364	TM4SF19	NM_138461.2	2,2628	1,1781	5,34E-15	5,16E-11	11,21134	10,03325	GGTGTTTTCA	Homo sapiens transmembrane 4 L six family member 19 (TM4SF19), mRNA.
UU	ILMN_172921	CRYAB	NM_001885.1	2,1445	1,1007	5,58E-12	2,93E-08	8,64361	7,54295	GTCCTCACTG	Homo sapiens crystallin, alpha B (CRYAB), mRNA.
U	ILMN_218886	GDF15	NM_004864.1	1,6255	0,7009	1,93E-10	5,32E-07	10,09842	9,39757	CCGGGGTGT	Homo sapiens growth differentiation factor 15 (GDF15), mRNA.
U	ILMN_181370	KIAA1199	NM_018689.1	1,5868	0,6661	6,06E-12	2,93E-08	9,29091	8,62483	GCAACGCTCC	Homo sapiens KIAA1199 (KIAA1199), mRNA.
U	ILMN_172124	KRT75	NM_004693.2	1,5376	0,6207	1,09E-10	4,22E-07	8,01681	7,39616	CTATACCCAT	Homo sapiens keratin 75 (KRT75), mRNA.
U	ILMN_179825	UPP1	NM_003364.2	1,5096	0,5942	5,09E-10	1,23E-06	9,25638	8,66222	ATCAAGAAG	Homo sapiens uridine phosphorylase 1 (UPP1), transcript variant 1, mRNA.
U	ILMN_225216	UBC	NM_021009.3	1,5066	0,5913	1,90E-10	5,32E-07	7,05602	6,46476	TCCAGAGAG	Homo sapiens ubiquitin C (UBC), mRNA.
U	ILMN_205878	IFI27	NM_005532.3	1,4863	0,5717	7,85E-05	0,0240809	8,21818	7,64646	CCAAGTTCAT	Homo sapiens interferon, alpha-inducible protein 27 (IFI27), transcript variant 2, mRNA.
U	ILMN_180120	GPNMB	NM_0010053	1,4264	0,5124	1,62E-08	2,85E-05	6,99818	6,4858	CAATAACAG	Homo sapiens glycoprotein (transmembrane) nmb (GPNMB), transcript variant 1, mRNA.
U	ILMN_205233	MYOC	NM_000261.1	1,4037	0,4892	1,84E-09	3,95E-06	7,18173	6,69255	TTCTTGGGGT	Homo sapiens myocilin, trabecular meshwork inducible glucocorticoid response (MYOC), mRNA.
U	ILMN_165711	C14orf78	XM_0011324	1,3797	0,4643	5,34E-08	8,61E-05	9,07439	8,61007	CACACTGTG	PREDICTED: Homo sapiens chromosome 14 open reading frame 78 (C14orf78), mRNA.
U	ILMN_170096	C3orf59	NM_178496.2	1,3607	0,4444	9,70E-09	1,88E-05	11,08157	10,6372	TTTGGTGTGG	Homo sapiens chromosome 3 open reading frame 59 (C3orf59), mRNA.
U	ILMN_175806	RGS4	NM_005613.3	1,3288	0,4101	3,15E-07	0,00040645	8,01647	7,60634	CCTTTCAAAG	Homo sapiens regulator of G-protein signalling 4 (RGS4), mRNA.
U	ILMN_241233	AKR1C2	NM_001354.4	1,3283	0,4095	1,14E-07	0,00016973	7,08308	6,67355	CCTGCTTGGC	Homo sapiens aldo-keto reductase family 1, member C2 (dihydrodiol dehydrogenase 2; bile acid binding protein; 3-alpha hydroxysteroid dehydrogenase, type III) (AKR1C2), transcript variant 1, mRNA. XM_943424 XM_943425 XM_943
U	ILMN_171312	AKR1C3	NM_003739.4	1,308	0,3874	1,15E-05	0,00676418	8,33339	7,946	GCCGGTGACT	Homo sapiens aldo-keto reductase family 1, member C3 (3-alpha hydroxysteroid dehydrogenase, type II) (AKR1C3), mRNA.
U	ILMN_319083	CCRL2	NM_0011309	1,3013	0,3799	7,93E-07	0,00095875	7,58418	7,20423	GGCGATCCCA	Homo sapiens chemokine (C-C motif) receptor-like 2 (CCRL2), transcript variant 2, mRNA.
U	ILMN_180993	NDRG1	NM_006096.2	1,2931	0,3708	3,74E-05	0,0141765	10,46289	10,09207	CTTCCCTGGC	Homo sapiens N-myc downstream regulated gene 1 (NDRG1), mRNA.
U	ILMN_175112	HIST1H4H	NM_003543.3	1,2928	0,3705	5,86E-06	0,0039092	7,93402	7,56357	CGCACTCTTT	Homo sapiens histone cluster 1, H4h (HIST1H4H), mRNA.
U	ILMN_324290	HIST2H2AA4	NM_0010408	1,2892	0,3665	3,02E-07	0,00040645	7,9738	7,60733	CTACATGGCT	Homo sapiens histone cluster 2, H2aa4 (HIST2H2AA4), mRNA.
U	ILMN_172135	KRT6B	NM_005555.3	1,2857	0,3626	2,65E-06	0,00216622	6,66868	6,30608	CTCTTGCAGT	Homo sapiens keratin 6B (KRT6B), mRNA.
U	ILMN_324315	AHNAK2	NM_138420.2	1,2736	0,3489	5,69E-06	0,0039092	8,10297	7,75405	TTGGGTGACA	Homo sapiens AHNAK nucleoprotein 2 (AHNAK2), mRNA.
U	ILMN_172453	LY96	NM_015364.2	1,2665	0,3409	1,28E-06	0,00137918	6,76727	6,42641	TGGGAGCCCA	Homo sapiens lymphocyte antigen 96 (LY96), mRNA.
U	ILMN_175951	RND3	NM_005168.3	1,2628	0,3366	2,11E-06	0,00194716	9,56337	9,22673	GAAGTGTTGA	Homo sapiens Rho family GTPase 3 (RND3), mRNA.
U	ILMN_172333	SCGB2A2	NM_002411.2	1,2585	0,3317	1,44E-06	0,00147016	6,68504	6,35329	CTTTCTGCAA	Homo sapiens secretoglobin, family 2A, member 2 (SCGB2A2), mRNA.
U	ILMN_173968	LRRC6	NM_012472.3	1,2558	0,3286	2,87E-06	0,00222252	7,4172	7,08861	ACCAAGTGA	Homo sapiens leucine rich repeat containing 6 (LRRC6), mRNA.
U	ILMN_170178	IFIT3	NM_0010316	1,2557	0,3285	1,15E-06	0,00130472	7,12618	6,79769	GCCCCAACCT	Homo sapiens interferon-induced protein with tetratricopeptide repeats 3 (IFIT3), mRNA.
U	ILMN_233659	ACSS2	NM_0010765	1,2507	0,3227	2,32E-06	0,00204121	10,12928	9,80654	GTTTTCTTGC	Homo sapiens acyl-CoA synthetase short-chain family member 2 (ACSS2), transcript variant 2, mRNA.
U	ILMN_170872	H2AFJ	NM_177925.2	1,2484	0,3201	1,59E-05	0,00878542	8,51795	8,19789	GAGTTAAACA	Homo sapiens H2A histone family, member J (H2AFJ), transcript variant 1, mRNA.
U	ILMN_173207	HIST2H2BE	NM_003528.2	1,2478	0,3194	2,69E-06	0,00216622	7,68282	7,36338	CACTTACCAC	Homo sapiens histone cluster 2, H2be (HIST2H2BE), mRNA.
U	ILMN_168708	LOC402251	XM_377933.3	1,2432	0,314	0,00012491	0,0305754	10,02338	9,70938	ATGCATCATG	PREDICTED: Homo sapiens similar to eukaryotic translation elongation factor 1 alpha 2 (LOC402251), mRNA.
U	ILMN_173399	DHRS9	NM_005771.3	1,2429	0,3137	5,69E-06	0,0039092	7,34776	7,03409	GCCCATTCAA	Homo sapiens dehydrogenase/reductase (SDR family) member 9 (DHRS9), transcript variant 1, mRNA.
U	ILMN_176846	TCN1	NM_001062.3	1,241	0,3115	3,31E-05	0,0139171	8,50398	8,19248	TGAGTGGAG	Homo sapiens transcobalamin I (vitamin B12 binding protein, R binder family) (TCN1), mRNA.
U	ILMN_174547	IRF9	NM_006084.4	1,241	0,3115	8,70E-05	0,0243836	8,8859	8,57435	GTCCGTGATA	Homo sapiens interferon regulatory factor 9 (IRF9), mRNA.
U	ILMN_236788	GEM	NM_181702.1	1,2402	0,3106	2,07E-06	0,00194716	8,67133	8,36071	CCCCCATTGT	Homo sapiens GTP binding protein overexpressed in skeletal muscle (GEM), transcript variant 2, mRNA.
U	ILMN_169004	TM7SF2	NM_003273.2	1,2338	0,3032	6,46E-06	0,00416306	7,16233	6,85917	TGTCACACCT	Homo sapiens transmembrane 7 superfamily member 2 (TM7SF2), mRNA.
U	ILMN_181170	GRN	NM_002087.2	1,2338	0,3031	2,21E-05	0,0109415	8,43166	8,12858	GGCCTTCCCT	Homo sapiens granulin (GRN), mRNA.
U	ILMN_239946	VAV3	NM_006113.4	1,2337	0,303	8,07E-06	0,00503477	7,9274	7,62437	CTTTGTCTTG	Homo sapiens vav 3 guanine nucleotide exchange factor (VAV3), transcript variant 1, mRNA.
U	ILMN_241325	EWSR1	NM_013986.2	1,2333	0,3025	0,00017593	0,0373841	8,19519	7,8927	CCTGGACCTT	Homo sapiens Ewing sarcoma breakpoint region 1 (EWSR1), transcript variant EWS-b, mRNA.
U	ILMN_186204	LOC731042	XM_0011282	1,2318	0,3008	1,64E-05	0,00882486	7,73946	7,4387	GCCGGTCATG	PREDICTED: Homo sapiens hypothetical protein LOC731042 (LOC731042), mRNA.
U	ILMN_171419	ACSS2	NM_018677.2	1,2301	0,2987	5,48E-05	0,0173772	10,32299	10,02425	TGCCCCAGTC	Homo sapiens acyl-CoA synthetase short-chain family member 2 (ACSS2), transcript variant 1, mRNA.
U	ILMN_172425	GRN	NM_002087.2	1,222	0,2892	3,48E-05	0,0141765	8,76984	8,48065	AAGGCTCGA	Homo sapiens granulin (GRN), mRNA.
U	ILMN_169115	MT1A	NM_005946.2	1,2209	0,288	0,00019458	0,040028	12,01951	11,73156	TGCTGCTCCT	Homo sapiens metallothionein 1A (MT1A), mRNA.
U	ILMN_169744	TXNIP	NM_006472.2	1,219	0,2857	9,93E-06	0,00600047	9,16034	8,87466	CAGCCATAG	Homo sapiens thioredoxin interacting protein (TXNIP), mRNA.
U	ILMN_237486	ATF3	NM_0010406	1,2143	0,2802	1,99E-05	0,0104056	7,35882	7,07866	CTCCCAGCGT	Homo sapiens activating transcription factor 3 (ATF3), transcript variant 4, mRNA.
U	ILMN_239329	GK	NM_203391.1	1,212	0,2774	0,00017882	0,0375853	8,31671	8,03931	CGTATGGAGT	Homo sapiens glycerol kinase (GK), transcript variant 1, mRNA.
U	ILMN_209566	TMEM156	NM_024943.1	1,2107	0,2759	2,12E-05	0,0107996	8,82181	8,54595	AACCAAGAT	Homo sapiens transmembrane protein 156 (TMEM156), mRNA.
U	ILMN_177836	PYGB	NM_002862.3	1,2072	0,2716	4,60E-05	0,0156774	10,73274	10,46111	GGGAACAGG	Homo sapiens phosphorylase, glycogen; brain (PYGB), mRNA.
U	ILMN_229776	KCNMA1	NM_002247.2	1,2049	0,2689	2,54E-05	0,0116867	9,56331	9,29439	GGTTCTGCAT	Homo sapiens potassium large conductance calcium-activated channel, subfamily M, alpha member 1 (KCNMA1), transcript variant 2, mRNA.
-	ILMN_173942	IFIT2	NM_001547.4	1,1929	0,2544	0,00012428	0,0305754	7,92207	7,66762	GCTGACCCA	Homo sapiens interferon-induced protein with tetratricopeptide repeats 2 (IFIT2), mRNA.
-	ILMN_171350	NPC1	NM_000271.3	1,1922	0,2536	0,00015628	0,0358498	10,01759	9,76399	CTTTGGCAAG	Homo sapiens Niemann-Pick disease, type C1 (NPC1), mRNA.
-	ILMN_177982	EDEM1	NM_014674.1	1,191	0,2521	5,46E-05	0,0173772	7,93749	7,68535	AGTGCTGGT	Homo sapiens ER degradation enhancer, mannosidase alpha-like 1 (EDEM1), mRNA.
-	ILMN_176897	HIST2H2AC	NM_003517.2	1,1909	0,2521	8,47E-05	0,0243836	8,16711	7,91505	CGCCCAGGG	Homo sapiens histone cluster 2, H2ac (HIST2H2AC), mRNA.
-	ILMN_177552	MAGED1	NM_0010053	1,1892	0,2499	3,08E-05	0,0135423	11,77157	11,52164	CAGCCAGTG	Homo sapiens melanoma antigen family D, 1 (MAGED1), transcript variant 3, mRNA.
-	ILMN_180124	IFITM1	NM_003641.3	1,1892	0,2499	0,0001698	0,0368924	7,60106	7,35111	GGCTGTTGCC	Homo sapiens interferon induced transmembrane protein 1 (9-27) (IFITM1), mRNA.
-	ILMN_176853	BHLHB2	NM_003670.1	1,1886	0,2492	2,96E-05	0,0133029	10,64359	10,39436	GGTCCAGAG	Homo sapiens basic helix-loop-helix domain containing, class B, 2 (BHLHB2), mRNA.
-	ILMN_206041	CD24	NM_013230.2	1,1876	0,248	3,71E-05	0,0141765	11,03986	10,79185	TAGACACTCC	Homo sapiens CD24 molecule (CD24), mRNA.
-	ILMN_1837428		AF131784	1,1838	0,2435	4,62E-05	0,0156774	10,35334	10,10986	TTCTATATGC	Homo sapiens clone 25194 mRNA sequence
-	ILMN_218189	BEX2	NM_032621.2	1,1832	0,2427	0,00016918	0,0368924	7,21495	6,97221	AGGGAGACC	Homo sapiens brain expressed X-linked 2 (BEX2), mRNA.
-	ILMN_167155	PHLDA2	NM_003311.3	1,1825	0,2418	8,21E-05	0,0243836	8,68747	8,44564	TGCGGGGCT	Homo sapiens pleckstrin homology-like domain, family A, member 2 (PHLDA2), mRNA.
-	ILMN_165149	HIST1H2BD	NM_138720.1	1,1821	0,2414	0,00012054	0,0305754	7,32314	7,08179	TGTGGATCCC	Homo sapiens histone cluster 1, H2bd (HIST1H2BD), transcript variant 2, mRNA.
-	ILMN_177428	CFB	NM_001710.4	1,1815	0,2406	4,34E-05	0,0155286	6,83989	6,59928	GTCCCTATGC	Homo sapiens complement factor B (CFB), mRNA.
-	ILMN_213608	MTE	NM_175621.2	1,1801	0,239	0,0002352	0,0459391	7,91209	7,67313	CCGTGGGCT	Homo sapiens metallothionein E (MTE), mRNA.
-	ILMN_179472	KRT20	NM_019010.1	1,1783	0,2367	0,00015341	0,0357417	6,66517	6,42849	CCAGGAAGA	Homo sapiens keratin 20 (KRT20), mRNA.
-	ILMN_168932	SCD	NM_005063.4	1,1773	0,2355	0,00013566	0,0323858	11,44546	11,20994	TAGGAATGT	Homo sapiens stearoyl-CoA desaturase (delta-9-desaturase) (SCD), mRNA.
-	ILMN_175313	LOC124220	NM_145252.1	1,1743	0,2319	5,34E-05	0,0173772	7,01837	6,78652	TTTGGGAAG	Homo sapiens similar to common salivary protein 1 (LOC124220), mRNA.
-	ILMN_211543	RAB32	NM_006834.2	1,1737	0,2311	6,47E-05	0,0201774	9,43966	9,20854	CCAGTGTTGC	Homo sapiens RAB32, member RAS oncogene family (RAB32), mRNA.
-	ILMN_221355	TMED10P	NR_002807.1	1,173	0,2302	0,00012701	0,0307005	10,39707	10,1669	CCAAAGTCA	Homo sapiens transmembrane emp24-like trafficking protein 10 (yeast) pseudogene (TMED10P), non-coding RNA.
-	ILMN_235555	PSAP	NM_002778.2	1,1724	0,2295	9,76E-05	0,0262137	11,2867	11,05717	TTCTGGTTGC	Homo sapiens prosaposin (PSAP), transcript variant 1, mRNA.
-	ILMN_178860	WBP2	NM_012478.3	1,1718	0,2287	9,10E-05	0,0251399	10,89672	10,668	GCGCTAACAT	Homo sapiens WW domain binding protein 2 (WBP2), mRNA.
-	ILMN_175597	ALDOC	NM_005165.2	1,1673	0,2231	0,00011199	0,0288733	8,33449	8,11136	CCATGATGA	Homo sapiens aldolase C, fructose-bisphosphate (ALDOC), mRNA.
-	ILMN_233726	PKIB	NM_032471.4	1,1673	0,2231	0,00020716	0,0412973	9,78699	9,56385	CCGCCGGAAT	Homo sapiens protein kinase (cAMP-dependent, catalytic) inhibitor beta (PKIB), transcript variant 3, mRNA.
-	ILMN_327577	LOC642567	XR_038054.1	1,1669	0,2227	0,00024504	0,0473839	7,50925	7,28656	AGGGAACAT	PREDICTED: Homo sapiens misc_RNA (LOC642567), miscRNA.
-	ILMN_1845037		BC031266	1,1659	0,2215	0,00017433	0,0373841	7,43932	7,21783	gccccctaattga	Homo sapiens cDNA clone IMAGE:5277162
-	ILMN_173686	TMEM140	NM_018295.2	1,1609	0,2153	0,00019901	0,0400864	6,74151	6,52621	ACCTGAAGC	Homo sapiens transmembrane protein 140 (TMEM140), mRNA.
-	ILMN_179419	CCPG1	NM_020739.2	1,1563	0,2096	0,00019169	0,0398569	8,20621	7,99664	TGTGGGTTCA	Homo sapiens cell cycle progression 1 (CCPG1), transcript variant 2, mRNA.
-	ILMN_323531	LOC92659	NR_015454.1	-1,1642	-0,2194	0,0001652	0,0367188	7,31049	7,52989	AGCTGGAGT	Homo sapiens hypothetical LOC92659 (LOC92659), non-coding RNA.
-	ILMN_168980	MRTO4	NM_016183.3	-1,1667	-0,2224	0,00015944	0,0358498	7,93236	8,15478	GGACCATCA	Homo sapiens mRNA turnover 4 homolog (S. cerevisiae) (MRTO4), mRNA.
-	ILMN_210633	H3F3A	NM_002107.3	-1,1703	-0,2269	0,00021682	0,0427829	11,61229	11,83917	TACAGGCCT	Homo sapiens H3 histone, family 3A (H3F3A), mRNA.
-	ILMN_181111	LOC400986	XM_0011268	-1,1708	-0,2274	9,44E-05	0,0257193	6,92824	7,15569	GACAGTGAC	PREDICTED: Homo sapiens protein immuno-reactive with anti-PTH polyclonal antibodies (LOC400986), mRNA.
-	ILMN_169174	KHDRBS3	NM_006558.1	-1,1765	-0,2345	0,00015124	0,0356648	9,52562	9,76013	AGGCACCTTC	Homo sapiens KH domain containing, RNA binding, signal transduction associated 3 (KHDRBS3), mRNA.
-	ILMN_239082	THOC2	NM_020449.2	-1,1837	-0,2433	5,04E-05	0,0168024	8,40608	8,6494	GAGAAGATG	Homo sapiens THO complex 2 (THOC2), transcript variant 2, mRNA.
-	ILMN_207533	HIST1H4C	NM_003542.3	-1,1879	-0,2484	0,00010751	0,0284064	12,38774	12,6361	TAGTATATGC	Homo sapiens histone cluster 1, H4c (HIST1H4C), mRNA.
-	ILMN_177089	YY1	NM_003403.3	-1,1905	-0,2515	3,73E-05	0,0141765	10,68948	10,94101	AGACCCTTCT	Homo sapiens YY1 transcription factor (YY1), mRNA.
-	ILMN_181302	CBX5	NM_012117.1	-1,1953	-0,2574	3,61E-05	0,0141765	8,47186	8,72928	TCAGAGGAT	Homo sapiens chromobox homolog 5 (HP1 alpha homolog, Drosophila) (CBX5), mRNA.
-	ILMN_328680	LOC10013113	XR_037336.1	-1,1973	-0,2597	0,00010871	0,0284064	8,37213	8,63187	TGGCGTTATG	PREDICTED: Homo sapiens misc_RNA (LOC100131139), miscRNA.
D	ILMN_324502	WASH1	NM_182905.3	-1,2047	-0,2687	3,90E-05	0,0145095	6,68328	6,95197	CTTGAGGCCA	Homo sapiens WAS protein family homolog 1 (WASH1), mRNA.
D	ILMN_167881	HAPLN1	NM_001884.2	-1,213	-0,2785	0,00025698	0,049201	8,87882	9,15736	AAATTCTCGG	Homo sapiens hyaluronan and proteoglycan link protein 1 (HAPLN1), mRNA.
D	ILMN_171057	PAPD5	NM_0010402	-1,2132	-0,2788	8,27E-05	0,0243836	7,68364	7,96245	GTGCAATGAT	Homo sapiens PAP associated domain containing 5 (PAPD5), transcript variant 2, mRNA.
D	ILMN_176958	PRSS35	NM_153362.1	-1,2236	-0,2912	8,62E-05	0,0243836	7,82514	8,1163	CAGCTCATGC	Homo sapiens protease, serine, 35 (PRSS35), mRNA.
D	ILMN_168735	MRPS23	NM_016070.2	-1,2288	-0,2973	0,00012261	0,0305754	8,86961	9,16691	GAAGTTCCA	Homo sapiens mitochondrial ribosomal protein S23 (MRPS23), nuclear gene encoding mitochondrial protein, mRNA.
D	ILMN_324072	LOC645233	NR_024382.1	-1,2358	-0,3055	4,08E-05	0,014888	7,28096	7,58646	GCGGAGAAC	Homo sapiens thymine-DNA glycosylase pseudogene (LOC645233), non-coding RNA.
D	ILMN_221499	LRRFIP1	NM_004735.2	-1,2375	-0,3074	2,42E-05	0,0116867	7,49117	7,79861	TGAGCAGGG	Homo sapiens leucine rich repeat (in FLII) interacting protein 1 (LRRFIP1), mRNA.
D	ILMN_178233	TDG	NM_003211.3	-1,2391	-0,3093	8,60E-05	0,0243836	8,01966	8,32896	TGAATGAAC	Homo sapiens thymine-DNA glycosylase (TDG), mRNA.
D	ILMN_175103	ITPRIPL2	NM_0010348	-1,2394	-0,3097	4,46E-05	0,0156774	9,25339	9,56304	CTGCAGGGA	Homo sapiens inositol 1,4,5-triphosphate receptor interacting protein-like 2 (ITPRIPL2), mRNA.
D	ILMN_174233	KCTD12	NM_138444.3	-1,2458	-0,3171	0,0001587	0,0358498	7,45917	7,77625	CTTGACATGA	Homo sapiens potassium channel tetramerisation domain containing 12 (KCTD12), mRNA.
D	ILMN_170841	GNL3L	NM_019067.4	-1,2501	-0,322	0,00019675	0,0400473	8,20891	8,53095	CCAAGCCCTG	Homo sapiens guanine nucleotide binding protein-like 3 (nucleolar)-like (GNL3L), mRNA.
D	ILMN_178573	TNFAIP6	NM_007115.2	-1,274	-0,3494	1,25E-05	0,00712801	8,63369	8,98305	CGATGATGT	Homo sapiens tumor necrosis factor, alpha-induced protein 6 (TNFAIP6), mRNA.
D	ILMN_322781	LOC729423	XM_0017269	-1,3134	-0,3933	2,51E-05	0,0116867	8,83958	9,23289	GGCCCCTATG	PREDICTED: Homo sapiens similar to Heterogeneous nuclear ribonucleoprotein A1 (Helix-destabilizing protein) (Single-strand RNA-binding protein) (hnRNP core protein A1) (HDP) (LOC729423), mRNA.
D	ILMN_169319	PI3	NM_002638.2	-1,3489	-0,4317	5,26E-06	0,0039092	7,44064	7,87237	CTGACTGCCC	Homo sapiens peptidase inhibitor 3, skin-derived (SKALP) (PI3), mRNA.
D	ILMN_211513	MSMP	NM_0010442	-1,3993	-0,4847	3,16E-05	0,0135871	9,63801	10,12271	GTTTCCATTG	Homo sapiens microseminoprotein, prostate associated (MSMP), mRNA.
